# Gut dysbiosis in cancer immunotherapy: microbiota-mediated resistance and emerging treatments

**DOI:** 10.3389/fimmu.2025.1575452

**Published:** 2025-08-25

**Authors:** Liza Eiman, Khadija Moazzam, Sumaira Anjum, Humera Kausar, Elham Abdullatif M. Sharif, Wisam Nabeel Ibrahim

**Affiliations:** ^1^ Department of Biotechnology, Kinnaird College for Women, Lahore, Pakistan; ^2^ Department of Biomedical Sciences, College of Health Sciences, QU Health, Qatar University, Doha, Qatar

**Keywords:** gut dysbiosis, cancer, microbiota, immunotherapy, tumor microenvironment, therapeutic potential

## Abstract

Cancer is a multifaceted disease driven by a complex interplay of genetic predisposition, environmental factors and lifestyle habits. With the accelerating pace of cancer research, the gut microbiome has emerged as a critical modulator of human health and immunity. Disruption in the gut microbial populations and diversity, known as dysbiosis, has been linked with the development of chronic inflammation, oncogenesis, angiogenesis and metastasis. This review discusses the microbial species associated with various types of cancer and the pathways involved in their tumorigenic effect including mechanisms like inflammatory cytokine response, immune modulation, genotoxicity and modification of the tumor microenvironment. Diagnostic tools such as metagenomics, metabolomics, and the use of dysbiosis indexes help in the detection of gut bacterial imbalances, enabling early detection of cancer and potential intervention. Gut dysbiosis diminishes the efficacy of cancer treatments including immunotherapies, and creates immunotherapy resistance by altering drug metabolism and driving immunometabolic reprogramming, allowing tumor cells to evade immune attack. Immunometabolic reprogramming through gut microbiota modulation provides a new avenue to be explored that can restore anti-tumor immunity and reverse resistance to cancer treatments. This review also highlights the use of fecal microbiota transplantation and probiotics to mitigate chances of dysbiosis-related cancer progression. Through a comprehensive assessment of the role of gut microbiota in cancer, this review underscores the need for the use of gut microbial biomarkers for cancer detection and microbiome-targeting strategies to individualize cancer treatment.

## 1 Introduction

The human body is a bustling metropolis of microscopic inhabitants who work silently to keep us healthy. These unsung workers are the gut microbiota, with 100 trillion microbes, including bacteria, fungi, viruses, protozoa, and archaea, residing in the mucosal surfaces along the host gastrointestinal (GI) tract (1,2). Most of these are obligate anaerobes that have evolved to exist in symbiosis with humans (3). Dysbiosis refers to a condition characterized by a loss of microbial diversity, proliferation of pathobionts, or a decrease in commensal microorganisms (4, 5).

Considerable evidence suggests that microbial imbalance in the gut is associated with inflammatory gastrointestinal disorders such as celiac disease, irritable bowel syndrome (IBS), inflammatory bowel syndrome, chronic inflammatory diseases such as asthma and allergies (6), and systemic disorders including neurological (7), hepatic (8), pancreatic (9), dermatological (10) and even cancer (11). Alterations in the microbiome are not only implicated in cancer development but can also impact cancer progression, treatment response, and susceptibility (12, 13).

Emerging evidence has highlighted the influence of gut microbiota on the effectiveness of immune checkpoint inhibitor therapies and development of treatment-related side effects (14–17). By exploring immune-microbiota interactions, we could uncover strategies to enhance the efficacy of immunotherapeutic approaches. Herein, we aimed to review the role of gut dysbiosis in oncogenesis, the underlying mechanisms for this process, and the therapeutic approaches that make use of this host-microbiota communication.

## 2 Gut microbiota

### 2.1 Composition and diversity

The human GI tract harbors a remarkable number of microorganisms – more than 10^14^ in number – so much so that they can be considered an accessory organ or a second gene pool (12, 18). In the human body, there are ten times more bacterial cells than human cells, and bacterial genetic information exceeds that of humans by more than a hundredfold. More recent studies estimated the human-to-bacterial cell ratio to be 1:1 (19). While the human genome contains approximately 27000 genes, the genomic content of our microbiome contains more than 33 million genes (3).

The alimentary canal alone is known to host more than 1000 different species of bacteria belonging to 100 distinct genera, approximately 160 of which can be present in a single individual (20). Of the 50 phyla of bacteria observed in the GI tract, the two most prevalent and conserved phyla were *Firmicutes* and *Bacteroidetes* but smaller proportions of *Fusobacteria*, *Verrucomicrobia*, *Cyanobacteria* and *Actinobacteria* were also present (21). There is significant variability in the microbiota composition at the species level among individuals, primarily because of functional redundancy, allowing bacteria from different species to form similar metabolites and perform conserved functions in the host digestive tract (22, 23).

All these insights into gut microbiota, their genetic and metabolic profiles, and composition have been made possible with advancements in metagenome sequencing. One of the most widely used methods for this purpose is targeting a gene present in all archaea and bacteria, the16S ribosomal RNA gene, and using its nine variable regions to differentiate between different species. However, whole-genome shotgun metagenomic sequencing can provide more reliable results (23). These techniques have facilitated the establishment of the Human Microbiome Project (HMP) and Metagenomics of the Human Intestinal Tract (MetaHit) project as sources of comprehensive data on the human microbiome (24, 25).

### 2.2 Physiological roles in host health

The gut microbiota provides a range of physiological benefits to its host, with digestion and metabolism being the most evident. Energy from the food we eat is made available to the body by microbes lining the gut, which produce enzymes that digest complex carbohydrates, otherwise indigestible by humans, into short-chain fatty acid (SCFA) metabolites such as butyrate, ~acetate, and propionate (23). After being absorbed into the gut epithelial layer, SCFAs play crucial roles in pathways related to cell proliferation, apoptosis, chemotaxis, and gene expression (23, 26). Microbial metabolites, such as butyrate, have anticancer properties, and the loss of these metabolites has been linked to certain types of cancer (23). Humans also need their microbiome to synthesize essential vitamins such as vitamins K and B (thiamine, riboflavin, nicotinic acid, pantothenic acid, pyridoxine, and biotin), which the host cannot produce on its own (23). Moreover, dysbiosis is linked to the development of type 2 diabetes and obesity owing to the role of microbiota in glucose and lipid metabolism (27).

Another key role of gut microbes in their hosts is the development of immunity. They help regulate anti-inflammatory pathways to prevent autoimmune diseases, and an imbalance in their composition can lead to inflammatory diseases (23). The microbiota in the GI tract acts as an epithelial barrier that prevents pathogens and toxins from entering the bloodstream. Beneficial gut bacteria compete with pathogenic microorganisms for nutrients and attachment sites, producing antimicrobial compounds and reducing the ability of pathogens to proliferate (28).

Gut microbiota maintains bidirectional communication with the host brain. While the brain can control mucin production, peristalsis, secretions and digestion in the gut, the microbial population can affect the host stress response by modulating the hypothalamic-pituitary-adrenal (HPA) axis (29). Gut bacteria can produce neuroactive compounds such as serotonin, dopamine, gamma-aminobutyric acid (GABA), and norepinephrine, which influence brain function and behavior (30).

## 3 Gut dysbiosis: causes and consequences

### 3.1 Factors influencing gut microbial balance

#### 3.1.1 Diet

Factors influencing microbial balance begin early in life, with diet being a key determinant starting from the infant feeding stage. During this period, a newborn’s immune system is not fully developed, and numerous studies have shown that the initial composition of microbiota in the gut is linked to, and therefore important in building mucosal immunity (31). Babies that are breast-fed have a higher composition of *Lactobacillus* and *Bifidobacterium* in the gut than those fed formula milk, in which the species *Enterococcus, Enterbacteria, Bacteroides, Clostiridia, and Streptococcus* dominate (32, 33). *Lactobacillus* and *Bifidobacterium* cause the breakdown of oligosaccharides in breast milk and the production of short-chain fatty acids (SCFAs), which in turn boost immune responses, such as the increased production of immunoglobulin G (IgG) and immunoglobulin A (IgA). Certain bacterial strains in the breastfed infant gut have a role in promoting the activity of T-cells and stimulating natural killer (NK) cells, CD4+, and CD8+ T cells, as well as directing the production of cytokines (34). This early microbial balance is crucial and lays the groundwork for a stable microbiota composition in adults, in which diet continues to play a major role.

Adults mainly follow three types of diet: Western, Vegetarian and Mediterranean. A typical Western diet is characterized by a high intake of processed foods, sodium, refined sugars, and saturated fats, and a low intake of fruits and vegetables. The Mediterranean diet has a high intake of fruits, vegetables, legumes, nuts, and olive oil, with a balanced consumption of fish and dairy products. On the other hand, vegans eat an entirely plant-based diet that does not come from animals. Varied nutrient intake from each dietary pattern affects the microbial composition of the intestinal tract (35). Dietary patterns have undergone a major shift over the past century. The high-fiber, low sugar, and minimally processed food of the 20^th^ century has been largely replaced by ultra-processed foods (UPFs) and a diet high in sugars and saturated fats and low in fiber, in the 21^st^ century (36–38). Research suggests that increased UPF consumption is linked to cancer through mechanisms like chronic low-grade inflammation, changes in microbiota, insulin resistance and obesity (39, 40). Moreover, consumption of Mediterranean diet decreases the risk of developing colorectal, gastric, liver and breast cancer and their mortalities (37). The increased incidence of early-onset cancers since 1990s has been seen particularly in cancers related to the GI system such as colorectal cancer, indicating a potential role correlation of diet-associated gut microbial changes and cancer (36).

The population profile of microbes is largely determined by the amount of fermentable fibers or microbiota-accessible carbohydrates (MACs) consumed by an individual (41). High MAC consumption results in a greater population of bacteria that ferment these fibers and produce SCFAs, which serve as a primary energy source for colonocytes, helping maintain the integrity of the intestinal wall. SCFAs also direct immune pathways such as the activation of inteleukin-22 (IL-22) which prevents metabolic disturbances and diet-related obesity (42). Foods rich in non-digestible fiber inulin, such as garlic, onion, wheat, and chicory, lower the population of the *Bilophila* genus, which eases constipation-related issues and increases the populations of *Bifidobacterium fecale/adolescentis, Bifidobacterium longum, Bifidobacterium catenulatum*, and *Bifidobacterium bifidum* (42). Metabolic byproducts of *Bifidobacteria* can be utilized by other bacteria, inhibiting the growth of some bacteria and encouraging the growth of others. For example, *Lachnobacterium, Ruminococcus*, and *Coprococcus* numbers decline, while the levels of important butyrate-producing bacteria, such as *Anaerostipes hadrus* and *E. rectale*, decline (35). Additionally, eating foods high in fructans, such as onions, garlic, and bananas, is associated with a decrease in harmful or opportunistic bacteria such as *Desulfovibrio, Enterobacter*, and *Salmonella*. A high-fiber diet also supports the growth of *Firmicutes* as opposed to *Bacteroidetes* (41). These bacteria secrete enzymes that digest complex carbohydrates into monosaccharides along with SCFA byproducts. Increased production of SCFAs is associated with a healthy gut and a balanced microbiota composition.

Fats consumed in a large amount damage the intestinal lining, causing a “leaky gut” and the number of gram-negative lipopolysaccharide (LPS)-producing bacteria also increases, while causing a decrease in beneficial bacteria such as *Bifidobacteria* and certain types of *Firmicutes*. LPS passes through the lining into the bloodstream, leading to chronic low-grade inflammation, which not only impacts metabolic health and weakens the immune system, but also creates a gut environment that may not be supportive of microbial diversity (41–43). A high-fat diet also increases the number of harmful *Bilophila wadsworthia*, whose metabolic products (e.g., hydrogen sulfide) are known to inflame intestinal tissue and contribute to inflammatory bowel disease (43). In a six-month intervention that was carried out, the negative effects of saturated fats were confirmed in young adults, which showed gut microbiome imbalance and inflammation. On the other hand, those with a low-fat diet demonstrated high α-diversity and increased numbers of beneficial *Blautia* and *Faecalibacterium prausnitzii*, which is an anti-inflammatory agent (44). It can be inferred that bacterial changes may result from metabolic disruptions due to the high levels of lipids in the blood or the presence of unusually large amounts of fat reaching the colon. Fat is usually digested by bile acids in the small intestine, but these bile acids may escape to the colon, where gut bacteria convert them into secondary bile acids that are linked to colorectal cancer and other gastrointestinal diseases. Bile acids in the colon can selectively favor the growth of bile-tolerant bacteria (e.g., *Bilophila*), while reducing populations of bile-sensitive beneficial bacteria, disrupting the gut microbial balance (31). In contrast, intake of unsaturated fats, such as omega-3, leads to an increase in beneficial bacteria, such as *Bifidobacterium, Lactobacillus, Lachnospira, and Roseburia* (35).

Regarding a protein-rich diet, depending on the source of protein, timing of consumption, and whether there is adequate intake of carbohydrates and fiber, the effects on microbial diversity vary (45). Proteins are a major source of nitrogen for the growth of microbiota and contribute to the production of valuable by-products including SCFAs (31). The levels of *Alistipes*, *Bilophila*, and *Bacteroides* increased with a protein-rich diet, along with decreased levels of *Roseburia*, *Eubacterium rectale*, and *Ruminococcus bromii*. If the protein is sourced mainly from plants, the number of *Lactobacilli* and *Bifidobacteria* increases (42). However, protein should only be taken in moderate amounts as the end products of protein fermentation, such as ammonia, sulfide, amines, and indoles, are carcinogens and cytotoxins. Data shows that increased intake of protein sourced from animals may be linked to gut dysbiosis with a decrease in the number of SCFA-producing bacteria and increased risk of cardiovascular diseases (45).

The Mediterranean diet typically has a high polyphenol content, sourced from fruits and vegetables, such as berries, grapes, tea, and cocoa. Most polyphenols are broken down by microbiota in the colon to produce bioactive products (31). A diet rich in polyphenols not only helps regulate oxidative stress and enhances intestinal permeability but also combats age-related imbalances in the gut microbiota (45). Overall, foods rich in polyphenols have been associated with an increase in fecal *Bifidobacteria* numbers (35,42). Lastly, excessive consumption of food additives, such as sweeteners and refined sugars, can significantly affect the healthy gut microbiota balance. Refined sugars, particularly fructose, increase the number of pro-inflammatory bacteria in the gut and damage the intestinal barrier, contributing to metabolic disorders such as obesity and type 2 diabetes (35, 43).

#### 3.1.2 Lifestyle factors

Smoking is a lifestyle choice that has a significant impact on gut microbiome balance. Data from different studies show that smokers with Crohn’s disease have an increased number of *Bacteroides-Prevotella* and reduced numbers of beneficial *Collinsella, Enterorhabdus*, and *Gordonibacter* compared to non-smokers (31,35). In addition, it has been observed that refraining from smoking for just eight weeks can significantly reduce dysbiosis in the gut, increasing the populations of *Firmicutes* and *Actinobacteria*, and decreasing *Proteobacteria* and *Bacteroidetes* populations (46).

Stress, another non-dietary factor that causes gut dysbiosis, has an adverse effect on gut health by disrupting the gut-brain axis. This can alter colonic motor activity and lead to changes in the composition of gut microbiota. In a state of stress, there is a notable decrease in beneficial bacteria such as *Lactobacillus* and an increase in harmful bacteria, potentially leading to conditions such as irritable bowel syndrome (IBS). This bidirectional relationship means that stress not only influences the gut microbiota, but extends its effect on brain function and mood (31). Furthermore, sleep deprivation exacerbates these effects by increasing the levels of pro-inflammatory cytokines and altering microbial populations. In contrast, good sleep quality is linked to high levels of bacteria belonging to the phyla *Verrucomicrobia* and *Lentisphaerae* (35). Studies have shown that stress-related changes in the gut microbiota can correlate with mood disorders, highlighting the role of microbiomes such as *Lactobacillus* and *Bifidobacterium* in mood enhancement (47).

An active lifestyle compared to a sedentary one is more beneficial in terms of a healthy and diverse microbiota population in the gut, as supported by numerous studies (31). Obese individuals show increased numbers of *Firmicutes* and decreased numbers of *Bacteroidetes* which are said to contribute to their condition as a result of greater energy harvest (31). Several other studies have shown that there are significant differences in microbiome composition between athletes and non-athletes. The levels of beneficial bacteria *F. prausnitzii, Roseburia hominis*, and *A. muciniphila* were higher in active women than in inactive women (35).

#### 3.1.3 Geographical influences

When it comes to environmental influences, geography plays a major part in the composition of gut microbiota. For example, individuals living in Africa have a different microbial profile than those living in developed countries (31). A study comparing fecal microbiota of children from an African village (Burkina Faso) with European children showed a greater microbiome richness and diversity in the rural African children. Bacteria from genus *Prevotella* and *Xylanibacter* capable of hydrolyzing cellulose and xylan were abundant in the African samples and completely absent in European ones. This could be explained by their diet, which is polysaccharide and fiber-rich and low in animal protein and fats, unlike an urban western diet (48). It can be hypothesized that diverse gut microbiota protects people living in less developed countries from noninfectious colonic diseases and dysbiosis-driven cancers. For instance, high-income countries (Australia-New Zealand and Europe) have a higher incidence but lower mortality due to colorectal cancer than low-and middle-income countries (South-Central Asia, Western and Middle Africa) (49). Contrarily, the incidence of infection-induced cancers such as stomach cancer, is significantly higher in less developed countries (50, 51).

Travelling to foreign countries with a different environment also exposes individuals to unfamiliar bacteria, viruses, or parasites, especially if they consume contaminated food or water. This results in a disruption of the gut microbiota balance and causes short-term gastrointestinal problems, such as diarrhea, or long-term problems, such as IBS (52). Developing countries have particularly poor sanitary conditions, and travelling to these countries puts individuals at greater risk. Travelling also disrupts the body’s circadian rhythm, which in turn affects the gut microbiota, as it works in sync with the body’s internal clock (53). Since microbiota composition is linked to the circadian rhythm, this also means that regular eating habits and fasting periods that align with the natural cycles of the body support a healthy gut microbial balance, and any irregularities in the eating pattern will adversely affect this balance (42).

#### 3.1.4 Antibiotics

The contribution of antibiotics to gut dysbiosis depends on the class of antibiotics administered, antibiotic overuse or abuse, mode of action, resistance to antibiotics, dosage amount, and the period of exposure (41, 54). In all cases, antibiotic usage modifies the gut microbiota composition, which may also be reversed over time. Broad-spectrum antibiotics, such as ciprofloxacin, lead to a decrease in *Bifidobacterium* in the *Actinobacteria* phylum. The population of *Alistipes* also decreases, and that of *Bacteroides* increases. There is also a notable decrease in the number of *Firmicutes*. Clindamycin use lowers the diversity of *Bifidobacteriaceae* and *Lactobacillus* (33). Another study showed that oral antibiotic therapy with macrolides caused a shift in *Bacteroides* and *Bifidobacterium* (55). Moreover, in an intervention involving 12 adult males, a four-day course of prophylactic antibiotics (vancomycin, gentamicin, and meropenem) led to an increase in *Enterobacteria* and *Fusobacterium nucleatum* while reducing butyrate-producing bacteria such as *Faecalibacterium prausnitzii* and *Roseburia hominis* (54). However, the gut resumed its balance six months prior to use, confirming that the effects of antibiotics can be temporary, depending on the duration of usage and type of antibiotic.

Disruption of the gut microbial balance due to antibiotic intake can lead to a multitude of adverse effects. In one study, gut dysbiosis caused by antibiotic administration led to disruption of the Toll-like receptor 4 (TLR4) signaling pathway, resulting in a significant increase in peanut-specific IgE levels and heightened T_H_2 cytokine responses, both of which play a role in triggering allergic reactions (43, 56). Imbalances related to antibiotic intake also significantly dysregulate cellular and humoral immune responses, as confirmed by *in vivo* and ex vivo studies (55).

### 3.2 Consequences of gut dysbiosis

Gut dysbiosis disturbs the symbiotic relationship between the gut bacteria and the host, leading to inflammation, weakened immune function, and altered metabolic pathways. First, microbial imbalance can compromise the integrity of the intestinal lining through various mechanisms, such as the production of acetaldehyde by gut bacteria from the breakdown of ethanol as a result of alcohol consumption and mucolytic activity, which directly breaks down the protective mucus layer of the intestines. This weakened barrier allows lipopolysaccharides (LPS) from gram-negative bacteria such as *Enterobacteriaceae* to translocate across the intestinal wall, triggering inflammatory responses. *Enterobacteriaceae*, although typically present in small numbers, can proliferate during dysbiosis, exacerbating inflammation due to the pyrogenic properties of LPS, which can intensify chronic inflammatory diseases, such as ulcerative colitis, Crohn’s disease, and even autoimmune diabetes (57).

The metabolic pathways of the body, particularly glucose and lipid metabolism, are also affected by microbial imbalance in the gut and may contribute to obesity, insulin resistance, and other metabolic disorders (44). Moreover, a reduction in butyrate-producing bacteria, such as *Faecalibacterium prausnitzii* and *Roseburia homini*s, during dysbiosis diminishes SCFA production, which can further weaken the gut barrier and impair immune function. Dysbiosis also increases the conversion of choline to trimethylamine (TMA), which is then converted to trimethylamine N-oxide (TMAO), a compound associated with cardiovascular diseases (57).

## 4 Gut dysbiosis and cancer

### 4.1 Mechanisms linking gut dysbiosis with carcinogenesis

#### 4.1.1 Role of microbial-induced inflammation

Gut dysbiosis involves a multitude of mechanisms and pathways that converge towards cancer. Microorganisms in the gut can initiate and progress cancer by activating inflammatory responses, reshaping the tumor microenvironment, compromising the gut barrier, and causing genotoxicity through carcinogenic metabolites.

Host-microbe interactions start at birth when the infant is exposed to microbes through the birth canal or from breast milk. The constant crosstalk between the gut microbiota and the host immune system maintains homeostasis of both innate and adaptive immunity (58, 59). The gut microbiota strikes a balance between pro-inflammatory (TNF-α, IL-1β, IL-2, IL-6, IL-15, IL-21, and IL-23) and anti-inflammatory (IL-10 and TNF- β) epithelial cytokine production (60, 61). The role of microbiota in the development of immune structures can also be deduced by observing a deficiency in immune cell populations, such as CD4+ T-cells, smaller size of Peyer’s patches, and fewer plasma cells producing IgA in germ-free animals (58).

**Figure 1:**
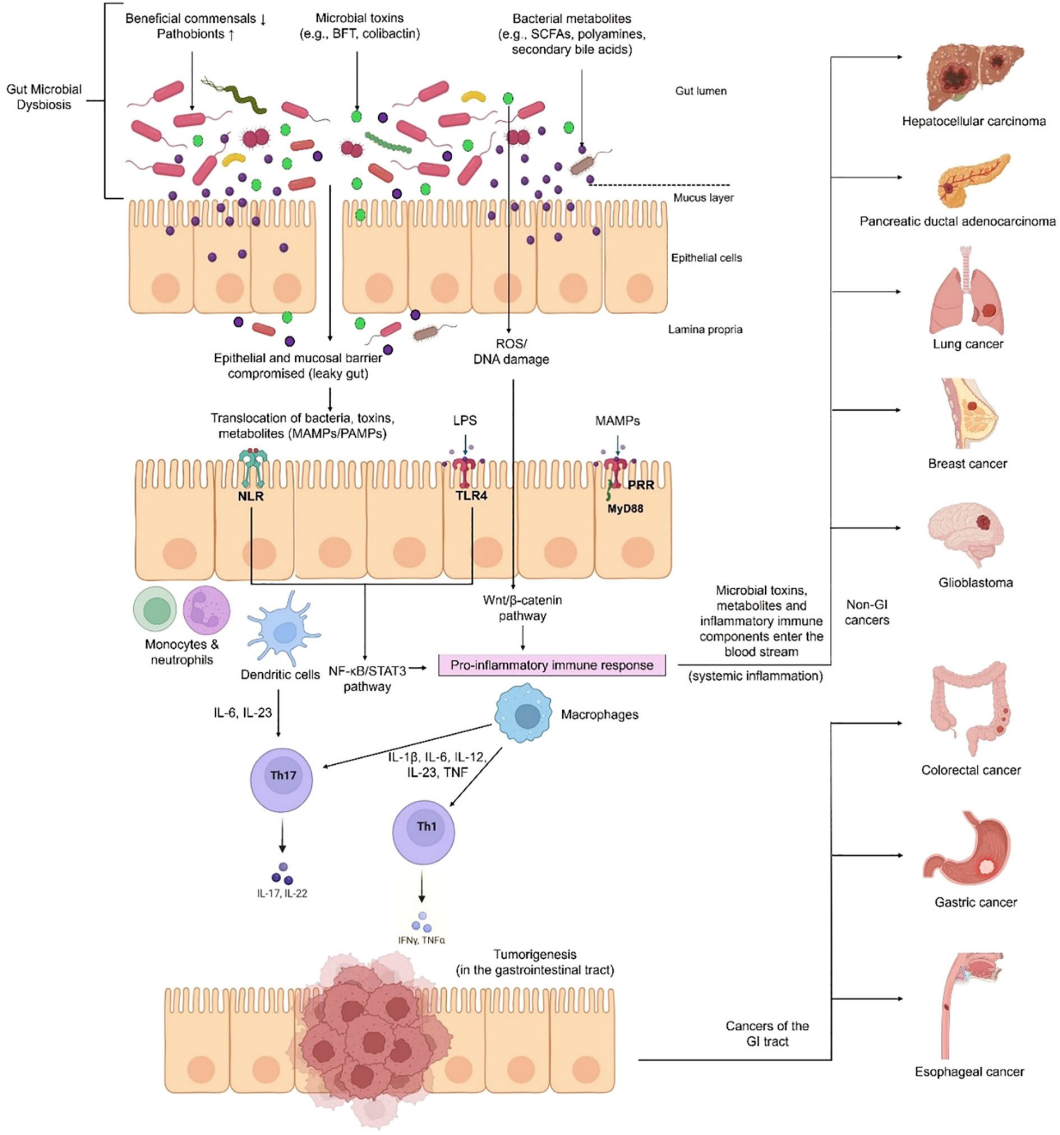
Mechanism of gut dysbiosis leading to inflammation and cancer initiation. Factors such as diet, lifestyle and antibiotic use can disrupt the delicate balance of beneficial and pathogenic gut microbes, leading to dysbiosis. This increases intestinal permeability, allowing pathogens, bacterial metabolites and toxins to move across the epithelial barrier. These microbial-associated molecular patterns (MAMPs) or pathogen- associated molecular patterns (PAMPs) are recognized by pattern recognition receptors (PRRs) such as Toll-like receptors (TLRs) and NOD-like receptors (NLRs) (e.g., TLR4 recognizes lipopolysaccharide (LPS) produced by certain bacteria). Inflammatory signaling cascades are triggered (such as NF-κB, STAT3 and Wnt/β-catenin pathways), promoting the release of inflammatory cytokines, and eventually leads to carcinogenesis (e.g., colorectal, gastric and oesophageal cancers). This inflammation can turn into systemic inflammation when pathogenic and immune components enter the blood stream and travel to other organs causing cancers like liver, pancreatic, lung, breast and brain cancers.

The host can identify microbial metabolites and structural constituents through microbe-associated molecular patterns (MAMPs) using pattern recognition receptors (PRRs) residing on the surfaces of immune and epithelial cells, such as Toll-like receptors (TLRs) and NOD-like receptions (NLRs) (62), as illustrated in **Figure 1** This leads to activation of signaling pathways that produce antimicrobial peptides (AMPs), such as C-type lectins, defensins, and cathelicidins, and initiate inflammatory responses (23). Microbial metabolites regulate host immunity; for example, SCFA and butyrate are responsible for the differentiation of regulatory T-cells (Tregs) (63). The normal function of T_H_17 (producing IL-17) and T_H_1 (producing IFNγ) cells is crucial for maintaining the epithelial barrier function of the gut microbiota, as these cells interact with microbial signals (58). Exposure to gut bacteria early in life helps train the immune system to respond appropriately to antigens and to maintain oral tolerance, potentially preventing allergies. Therefore, children with dysbiosis are prone to developing allergies (64).

Marshall and Warren’s discovery of *Helicobacter pylori* as the causative agent of stomach ulcers and gastric cancers in 1984 led to research on the role of bacteria in other types of cancers and their underlying mechanisms (65). Inflammation, the activity of the immune system usually used for host defense, is also required for tissue repair and homeostasis (66). Paradoxically, in the case of cancer, this defense mechanism can promote cancer progression and tumor growth instead of attacking the cancer (67). Inflammatory cells within the tumor microenvironment produce growth factors and cytokines that aid in cell proliferation, metastasis, and evasion of anti-tumor immune responses (13). Therefore, chronic inflammation has a well-established role in cancer, and since the gut microbiota extensively interacts with various aspects of the immune system, it is also involved in carcinogenesis.

Around 20% of human malignancies are associated with microbes (68). It is interesting to note that the 1-million-fold higher proportion of bacteria in the colon compared to the small intestine coincides with the fact that 12 times more cancers develop in the colon than in the small intestine (69). One might imply that a sterile environment would lower the risk of developing cancer in that organ. This, however, might not necessarily hold true because recent studies have challenged the idea of organs being completely sterile or pathogen-free. Microbes have been found to exist even in organs previously considered to be sterile such as brain, lungs, kidney, liver, pancreas, spleen and prostate (70–72). Studies have shown cancers of these organs to host intratumoral microbial signatures which can influence oncogenesis (73, 74).

The subsequent section highlights microbiota-cancer association across eight different cancer types, and is summarized in **Table 1**.

**Table 1:** Major representative gut microbial species associated with multiple cancer types.

Cancer type	Representative microbial species
Colorectal cancer (CRC)	*Fusobacterium nucleatum*, *Bacteroides fragilis*, *Peptostreptococcus anaerobius*, *Escherichia coli NC101*, *Prevotella intermedia*, *Parvimonas micra*, *Alistipes finegoldii*, *Porphyromonas asaccharolytica*, *Streptococcus gallolyticus*
Gastric cancer	*Helicobacter pylori*, *Parvimonas micra*, *Streptococcus anginosus*, *Dialister pneumosintes*, *Peptostreptococcus stomatis*, *Slackia exigua*, *Lactobacillus*, *Streptococcus*, *Lactococcus*, *Bifidobacterium*
Oesophageal cancer	*Akkermansia muciniphila*, *Enterobacteriaceae*, *Lactobacillus fermentum*, *Streptococcus* spp., *Campylobacter concisus*
Liver cancer (HCC)	*Bacteroides*, *Clostridium XIVa*, *E. coli*, *Ruminococcaceae*, *Lachnospiracea incertae sedis*
Pancreatic cancer (PDAC)	Increased *Proteobacteria*, Decreased *Firmicutes*, fungi like *Malassezia* spp.
Lung cancer	Increased *Enterococcus*, *Bacteroides*, *Prevotella*, *Veillonella*, *Fusobacterium*, Reduced *Bifidobacterium*, *Lachnospiraceae*
Breast cancer	Elevated *Clostridia*, specific estrobolome-related bacteria, increased *Blautia* sp.
Brain cancer (GBM)	Increased *Escherichia coli*, *Bacteroides vulgatus*, *Akkermansia*, *Fusobacterium*, Reduced *Lachnospira*, *Bifidobacterium*

##### 4.1.1.1 Colorectal cancer

The mechanisms leading from pathogen infection to inflammatory disease and cancer can include a genetic predisposition that makes the host conducive for inflammatory immune responses and weakens host epithelial defense due to environmental causes or microbial toxins (75). A low-fiber diet can cause dysbiosis by reducing the number of butyrate-producing bacteria and increasing the number of bacteria that can disrupt the integrity of the intestinal barrier, such as *Bacteroides caccae* and *Akkermansia muciniphila*. This enhances permeability, and susceptibility to pathogen entry can lead to inflammation and gastrointestinal carcinogenesis (76).

Microbial-induced inflammation by bacteria, such as *Bacterodies fragillis, Streptoccus bovis/gallolyticus* and *Escherichia coli NC101* has been associated with the development of colorectal cancer (77). Bacteria have various mechanisms to induce these inflammatory pathways in the host; for example, *Peptostreptococcus anaerobius* activates TLR2 and TLR4 pathways, enhancing the production of reactive oxygen species (ROS) and cholesterol biosynthesis, which lead to cell proliferation and colon cancer (78). Metagenome analysis of 526 samples from individuals of multiple ethnicities with colorectal cancer (CRC) showed an increase in bacterial species such as *Fusobacterium nucleatum, Thermanaerovibrio acidaminovorans, Bacteroides fragilis, Prevotella intermedia*, *Parvimonas micra*, *Alistipes finegoldii* and *Porphyromonas asaccharolytica* (79). Experiments using a mouse model of CRC demonstrated that *Peptostreptococcus anaerobius* can adhere to colonic tumor cells via its putative cell wall binding repeat 2 (PCWBR2) protein, which interacts with α_2_/β_1_ integrin, a receptor overexpressed in CRC cells. The subsequent signaling cascade eventually leads to the activation of NF-κB and an increase in pro-inflammatory cytokine levels (IL-10 and INF) in the tumor, thereby promoting carcinogenesis (**Figure 1**) (80).

In a dysbiotic gut environment, *Klebsiella* spp., which are usually present in the oral cavity and in small amounts in the gut, can translocate from the mouth to the gut. This colonization and multiplication of bacteria leads to inflammation by activating T helper 1 (T_H_1) cells (81). Chronic inflammation is a known cause of colon cancer (76). *E. coli* has been implicated in the pathogenesis of CRC by inducing irritable bowel disease (IBD) (82). A study on mice deficient in the NLRP6 inflammasome showed gut dysbiosis leading to inflammation and an increased risk of colorectal cancer compared to wild-type mice. Activation of the interleukin-6 (IL-6) pathway is responsible for inflammation and tumorigenesis via microbial-induced production of chemokine ligand 5 (CCL5). Mice lacking NLRP6 and ASC inflammasomes have also been found to contain colitogenic gut microbes transmissible to healthy mice (83). A similar study on TRUC mouse models (lacking T-bet and RAG2 genes) showed the importance of commensals in inflammatory responses using the MyD88-independent pathway that led to the development of colitis-associated colorectal cancer (caCRC). T-bet is a transcription factor expressed in immune cells that controls the commensal-host balance and the expression of cytokines and chemokines in the colon (84).

In addition to mouse models, gut dysbiosis has also been observed in humans with colorectal cancer. Sequencing and qPCR of bacterial DNA extracted from the stool samples of 179 patients after colonoscopy revealed higher levels of *Bacteroides/Prevotella* in patients with CRC than in those with normal colonoscopy. Immunohistochemical analysis showed elevated amounts of Interleukin-17 (IL-17)-producing cells within the intestinal mucosa of cancer patients, but not in normal ones (85).

Some bacteria have developed special cancer-causing mechanisms; for example, *Fusobacterium nucleatum* uses its virulence factor FadA to enter the colonic epithelial cells. This adhesin protein binds to E-cadherin in epithelial cells, activating the β-catenin signaling pathway and inflammatory genes, leading to tumor cell proliferation and colorectal cancer progression (86). When the gut barrier is compromised, microbial products can breach the epithelial barrier and trigger inflammation by activating the IL-23 and IL-17 pathways, leading to chronic inflammation and eventually, cancer, as depicted in **Figure 1**. The presence of T-helper interleukin (IL)-17-producing (T_H_17) cells and myeloid cells in the tumor microenvironment is typical of tumor-elicited inflammation and tumor growth in CRC (87).

##### 4.1.1.2 Gastric cancer

A multitude of studies have shown that *Helicobacter pylori* infections are indicative of gastric cancer. This link was established as early as the 1900s, when scientists concluded that seropositivity of IgG antibodies against *H. pylori* was associated with an increased risk of gastric adenocarcinoma (88). When the natural microbial balance of the stomach is altered, *H. pylori* can cause a series of changes that lead to cancer, such as atrophy (tissue damage), gastritis (chronic inflammation), dysplasia (abnormal cell growth), and intestinal metaplasia (the stomach lining starts to resemble the intestinal lining) (89). This occurs in three ways: by production of cytotoxins VacA and CagA, which trigger oncogenic pathways; by production of reactive oxygen species (ROS) that stimulate the inflammatory response; and by destroying parietal cells, causing increased acid production by compensatory enhancement of gastrin synthesis (76). This leads to the development of malignancy. The carcinogenic mechanism of *H. pylori*-induced gastric cancer involves epigenetic changes via the abnormal DNA methylation of epithelial cells in the stomach (90). Lipopolysaccharides (LPS) present on the surface of *H. pylori* act as ligands for Toll-like receptor 4 (TLR4), triggering the activation of downstream signaling molecules, including NF-κB, leading to inflammation and subsequent tumor formation (**Figure 1**) (91).

The GI microbiome also differs among the different stages of gastric cancer progression (92, 93). Compared to superficial gastritis, atrophic gastritis, and intestinal metaplasia, gastric cancer samples showed enrichment of five bacterial taxa (*Parvimonas micra, Streptococcus anginosus, Dialister pneumosintes*, *Peptostreptococcus stomatis* and *Slackia exigua*) (93). Dysbiotic spikes in the number of lactic acid bacteria such as *Lactobacillus, Streptococcus, Lactococcus* and *Bifidobacterium*, are also associated with gastric cancer (94).

##### 4.1.1.3 Esophageal cancer

Cancer due to changes in the microbiome is not exclusive to colon and gastric cancer. The presence of six bacterial phyla, Firmicutes, Actinobacteria, Fusobacteria, Bacteroidetes, Proteobacteria, and TM7, has been linked to healthy human esophagus (95). Studies have shown alterations in esophageal microbes and loss of diversity to cause Barrett’s esophagus (BE), gastroesophageal reflux disease (GERD), and eventually esophageal cancer. Gram-negative bacteria are known to cause esophageal inflammation through activation of the NF-κB pathway by LPS on the surface of these bacteria. This further induces IL-8 production, causing Barrett’s esophagus and increasing the risk of esophageal adenocarcinoma (EAC) (95, 96). A study demonstrated a decrease in *Veillonella* and an increase in *Akkermansia muciniphila* and *Enterobacteriaceae* populations in patients with esophageal adenocarcinoma (97). Higher than usual amounts of lactic acid-producing bacteria, such as *Lactobacillus fermentum* (98) and *Streptococcus* spp (99)., and gram-negative bacteria such as *Campylobacter concisus* (100) are signatures of EAC and inflammatory conditions such as BE and GERD, which can lead to cancer because these bacteria aid in glucose utilization by cancer cells, enhancing cancerous growth. Surprisingly, infection with *H. pylori*, a bacterium responsible for gastric ulcers and gastric cancers, tends to decrease the risk of esophageal adenocarcinoma (EAC). Although *H. pylori* does not normally reside in the esophageal mucosa, it can affect the microbial diversity of the lower esophagus and provide protection against EAC (95, 101).

##### 4.1.1.4 Liver cancer

Gut microbes and their metabolites can interact with the liver and impact liver immunity via the blood supply from the intestines and enterohepatic circulation (102). Perturbations of the gut microbiota have been linked with hepatocellular carcinoma (HCC); for example, studies have reported reduced numbers of butyrate-producing bacteria, higher numbers of lipopolysaccharide-producing bacteria (103, 104), and higher numbers of SCFA-producing bacteria in HCC patients (105). Moreover, investigation of the intestinal microbiota of patients with HCC caused by non-alcoholic fatty liver disease (NAFLD)-related cirrhosis revealed *Bifidobacterium* reduction, and Ruminococcaceae and *Bacteroides* abundance compared to healthy people. Activated inflammatory immune components, such as CCL3, CCL4, CCL5, IL-8, and IL-13 were also found in the HCC group (106).

Studies suggest that there is a greater richness of bacterial species in fecal samples of hepatitis B virus (HBV)-related hepatocellular carcinoma (HCC) than in the non-HBV related-HCC group (107). Other studies comparing the gut microbiomes of healthy people with those of HBV-related HCC patients found enriched levels of *Bacteroides*, *Clostridium XIVa*, *Lachnospiracea incertae sedis* (108), and *E. coli* (109) in HCC patients. Patients with HBV-related HCC have greater bacterial diversity than those with liver cirrhosis, but the pro-inflammatory cytokine IL-2 was higher in both groups when compared to healthy controls, suggesting a correlation between gut microbiome diversity and HCC (110). Activation of the NF-κB pathway in myeloid cells is linked to an increased risk of gastric cancer *by H. pylori* and an increased risk of hepatocellular carcinoma (HCC) to chronic hepatitis B and C virus infections (75). Dysbiosis activates the NF-κB and STAT3 pathways, the former expressing anti-apoptotic and pro-inflammatory genes and the latter having an oncogenic role, and their interaction is crucial for microbial-induced carcinogenesis (59).

##### 4.1.1.5 Pancreatic cancer

Gut microbiome alterations have also been observed in pancreatic cancers. For example, patients with pancreatic carcinoma have a unique microbial profile marked by decreased probiotic and butyrate-producing bacteria, increased lipopolysaccharide-producing bacteria, and reduced overall diversity (111). Metagenome sequencing of fecal samples from ductal adenocarcinoma (PDAC) patients showed an increase in bacteria belonging to the phylum Proteobacteria and a decrease in members of the phylum Firmicutes (112). The gut microbiome can influence the tumor microbiome and survival outcomes in pancreatic cancer, with higher microbial diversity being a positive factor for long-term survival (113). Researchers fed fluorescently labeled *Enterococcus faecalis* to wild-type mice to determine whether gut microbes can translocate to the pancreas, and the experiment yielded a positive result, confirming this hypothesis. Quantitative PCR analysis further revealed that there are more bacteria in pancreatic cancerous tissues than in normal tissues of mice and humans, indicating that the gut microbiome can translocate to the pancreas in cancer-susceptible mice and play a role in tumor development (114). Fungi show a similar behavior. Fungi migrate from the gut to the pancreas, and 3000 times higher numbers of fungi were found in cancerous pancreatic tissues of mice and humans compared to healthy pancreas. *Malassezia* spp. are enriched in pancreatic ductal adenocarcinoma (PDA) tumors, implicating fungi in tumorigenesis (115).

##### 4.1.1.6 Lung cancer

Interactions of the gut-lung axis have also been explored, and evidence has linked gut dysbiosis with lung cancer. As stated earlier, dysbiotic states interfere with the mucosal barrier function and promote mucosal permeability (leaky gut), allowing the translocation of microbes or their metabolites into the bloodstream. This triggers systemic inflammation that can spread to several organs including the lungs (116). When researchers sequenced 16S rRNA from fecal samples of 60 healthy and lung cancer (LC) patients, increased levels of *Enterococcus* were observed in the LC group, whereas elevated levels of the genus *Bifidobacterium* and phylum Actinobacteria were seen in the control group (117). Similar studies have linked elevation in *Bacteroides* and *Prevotella* levels and a decrease in *Lachnospiraceae* to lung cancer (116, 118). A rigorous study of 181 fecal samples found *Ruminococcus* was abundant in the cancer group (119). Higher levels of *Veillonella*, *Bacteroides* and *Fusobacterium*, and lower levels of *Kluyvera*, *Enterobacter*, *Fecalibacterium, Escherichia-Shigella* and *Dialister* along with signs of inflammation such as elevated serum levels of sCTLA-4, IL-17 and IL-6 have been observed in fecal samples of lung cancer patients compared to healthy people (120).

##### 4.1.1.7 Breast cancer

Gut microbiome disruptions affect estrogen metabolism by changing the levels of microbial metabolites and are associated with breast cancer through changes in the levels of anti-tumor metabolites. The collection of bacterial genes in the gut responsible for metabolizing estrogen, called the estrobolome, can influence the risk of developing estrogen receptor-positive breast cancer (121). A study conducted on postmenopausal women showed that elevated levels of *Clostridia* and a higher diversity of the gut microbiome were linked to an increased ratio of hydroxylated estrogen metabolites in the urine compared to parent estrogens (estrone and estradiol), indicating a greater risk of breast cancer (122). Comparison of the gut microbiome of postmenopausal women with breast cancer compared to healthy controls and premenopausal women showed an increased abundance of 38 bacterial species and a decreased abundance of seven bacterial species in the breast cancer group (123).

Gut microbiota differs between healthy women and those with breast cancer, as microbiota can affect estrogen metabolism and immune responses (124). The intestinal microbiome differs even among different stages of breast cancer. For example, in a study on fecal samples from breast cancer patients, *Blautia* sp. was present in significantly higher numbers in later clinical stages than in earlier stages (125). This is because under homeostatic conditions, certain gut microbes produce β-glucuronidase, an enzyme that deconjugates estrogen and allows it to be reabsorbed into the circulation. When dysbiosis occurs, changes in β-glucuronidase activity can cause higher levels of circulating estrogen in the body, which can aid the growth of ER-positive breast cancer cells (126, 127).

##### 4.1.1.8 Brain cancer

Microbes can interact with the brain along the microbiome-gut-brain axis and influence neuroinflammation and carcinogenesis because they can affect the synthesis or amounts of amyloid proteins, antioxidants, lipopolysaccharides, short-chain fatty acids, signaling molecules, cytokines, reactive oxygen species, and certain amino acids, all of which are involved in oncogenic or oncolytic processes, such as apoptosis, autophagy, activation of receptors and immune signaling pathways, and maintenance of DNA integrity (128, 129). Gut microbes can control tryptophan breakdown into kynurenine and other downstream metabolites, which can cross the blood-brain barrier and contribute to the inflammatory conditions of the brain and brain tumors (130, 131). Elevated kynurenine levels have been associated with immune suppression, as they reduce the activity of CD4^+^ T cells, which are crucial for the body’s anti-tumor response (128).

A study comparing fecal samples from glioblastoma (GBM) patients with those from healthy people showed a greater level of diversity in GBM samples. An increase in Proteobacteria and a decrease in Firmicutes were observed at the phylum level in GBM patients, but at the species level, *Escherichia coli* and *Bacteroides vulgatus* were present in significantly higher amounts (132). In contrast, another study showed a decreased microbial diversity of fecal bacteria in patients with brain tumors compared to that in healthy controls. There was a marked decline in probiotic bacteria (*Lachnospira* and *Bifidobacterium*) and an increase in pathogenic bacteria (*Proteobacteria* and *Fusobacteriota*) (133). The microbial composition also differs between benign and malignant brain tumors. Analysis of fecal specimens showed decreased gut microbial diversity in brain tumor patients compared to healthy individuals, an increased proportion of pathogenic bacteria (*Enterobacteriaceae*) in patients with benign meningioma, and an increased proportion of cancer-causing bacteria (*Akkermansia* and *Fusobacterium*) in malignant glioma patients (134).

#### 4.1.2 Microbial metabolism and genotoxicity

Inflammation-induced carcinogenesis pathways have been observed in colon cancer caused by enterotoxic *Bacteroides fragilis* which secretes a toxin that induces colitis by activating Signal Transducer and Activator of Transcription 3 (STAT3), a pathway involved in tumor formation. Pro-inflammatory cytokines are released and the T_H_17 immune response is initiated as a result of STAT3 activation, which is further enhanced by IL-23 (135). *Bacteroides fragilis* toxin (BFT) produced by *Enterotoxigenic B. fragilis* and polyketide synthase produced by *E. coli* are known factors that contribute to carcinogenesis in CRC (136–138).

Colitis-susceptible mice lacking the anti-inflammatory cytokine IL-10 were used to demonstrate the genotoxic effects of the commensal bacterium, *Escherichia coli NC101*. This bacterial strain contains a set of genes, polyketide synthase (pks) genotoxic island, allowing them to produce genotoxic compounds, such as colibactin, which induce double-stranded breaks in host cell DNA, leading to mutations, inflammation, and tumorigenesis (139). Furthermore, gut microbial dysbiosis caused by genetic or dietary obesity is involved in the development of liver cancer, primarily due to DNA damage caused by the gut microbial metabolite deoxycholic acid (DCA) (140).

Other bacterial metabolites such as SCFAs and polyamines, when released in abnormal amounts, have been seen to cause cancer by exerting toxic effects that lead to inflammation (138). For instance, hydrogen sulfide (H_2_S) produced by certain gut bacteria, such as *Escherichia coli, Enterobacter aerogenes, Salmonella enterica, Bacillus, Corynebacterium, Clostridia, Staphylococcus, Klebsiella* and *Rhodococcus* is known to cause DNA damage and inflammation in the gut epithelium, which may develop into colorectal cancer (41–44, 138).

Microbes residing in the GI tract can disrupt genomic stability and cause DNA damage in the host, which can lead to mutational events and cancer. Bacterial toxins such as colibactin, BFT, and cytolethal distending toxin (CDT) are known to cause double-stranded DNA breaks (141). Increased production of reactive oxygen species (ROS) by certain gut bacteria is known to cause oxidative DNA damage, genotoxicity, and ultimately, cancer (**Figure 1**) (121, 142). *Fusobacterium nucleatum, Escherichia coli, Actinobacillus actinomycetemcomitans, Shigella dysenteriae, Salmonella typhi, Helicobacter* spp.*, Campylobacter* spp., and *H. ducreyi* are known to be involved in DNA damage and tumorigenesis. Genetic predisposition of individuals and polymorphisms in their base excision repair (BER) genes can affect their ability to repair microbial-induced DNA lesions, making some people more susceptible to developing colon cancer (141).

### 4.2 Influence on tumor microenvironment and immune evasion

#### 4.2.1 Interactions with immune system

Different cell populations, such as endothelial cells, tumor invading immune cells [e.g., myeloid-derived suppressor cells (MDSCs) and tumor-associated macrophages (TAMs)], immunosuppressive cytokines of the extracellular matrix, and abnormal blood vessel systems make up the tumor microenvironment (TME) (143). The TME plays a clear role in tumor invasion, tolerance, and proliferation. The gut microbiota interacts with the immune system and works in sync to produce a modulatory effect on the tumor microenvironment. Some microbes and their metabolites have a positive effect, that is, they recruit immune cells and activate metabolic pathways that result in tumor reduction. However, many microbes and their metabolic products have been noted to modulate the tumor microenvironment in such a way as to promote metastasis and the growth of tumors. This primarily occurs during dysbiosis, when harmful microbes outnumber beneficial ones or microbial diversity is reduced. For example, when the diversity is low, the number of neutrophils in the blood increases which are activated by toll-like receptor-4 (TLR4) receptors, which direct the migration of cancer cells to nearby endothelial cells. Neutrophils further mediate the adhesion of cancer cells to the vasculature through neutrophil Mac-1/ICAM-1. This causes cells to enter the bloodstream, resulting in metastasis. The pro-inflammatory cytokines and chemokines released by activated neutrophils also contribute to a tumor-promoting microenvironment (144).

Similarly, *F. nucleatum* recruits MDSCs and TAMs to the microenvironment, where they exert their immunomodulatory effects in colorectal cancer (CRC) (143). Tumor-associated macrophages influence T cell activity, resulting in immunosuppression and metastasis. High *F. nucleatum* levels promote macrophage polarization to the M2 phenotype by activating the NF-κB pathway. M2 macrophages drive tumor progression and metastasis. The levels of pro-inflammatory cytokines (e.g., different interleukins, C-X-C motif chemokine ligand 1 (CXCL1), tumor necrosis factor (TNF)-α, and interferon (IFN)-γ) in the TME increase as a result of harmful bacteria in the gut. This produces a heightened inflammatory response that also plays a role in CRC progression (145).

Gram negative bacteria in the gut release outer membrane vesicles (OMVs) as part of their normal physiology which contain components like lipopolysaccharides, nucleic acids, proteins and phospholipids, e.g., *Bacteroides fragilis* releases OMVs containing polysaccharide A and *Helicobacter pylori* releases OMVs with VacA. These are required for intercellular communication, delivering signaling molecules to target cells and interacting with the host immune system. However, dysbiosis and change in the gut environment can cause bacteria to increase OMV production (146–148). These vesicles can communicate with host cells within the TME, thereby influencing the immune response. Specifically, OMVs have been shown to drive a shift in the TME toward a pro-T_H_1 immune profile, characterized by the upregulation of key cytokines, such as CXCL10 and interferon-gamma (IFN-γ), which are associated with enhanced anti-tumor immunity (149).

#### 4.2.2 Influence on angiogenesis and tissue remodeling

Angiogenesis translates to the process of generating new blood vessels from existing ones, and although it is a necessary physiological process for wound healing and development, when seen in the context of tumor progression, it becomes a pathological process. Here, the existing vascular basement membrane is degraded, and the extracellular matrix (ECM) undergoes modification, which drives endothelial cell migration and invasion in neighboring tissues (150). Pathogenic angiogenesis is triggered by chronic inflammation or cancer, which develop as a result of gut dysbiosis (151). In tumors, angiogenesis facilitates sustained growth by supplying oxygen and nutrients to proliferating cells, and the gut microbiota plays a pivotal role in this.

The inflammatory microenvironment, influenced by dysbiotic microbial factors, accelerates tumor angiogenesis. Gut microbes can influence the vascular network by selectively activating mucosal endothelial and mesenchymal cells through toll-like receptors (TLR) and NOD-like receptor pathways (151). These pathways foster specific angiogenic responses leading to increased vascularization, proliferation, and tube formation. Microbial metabolites of different bacteria further drive this process, contributing to an enhanced proangiogenic state. Tissue factor (TF), a membrane receptor activated by inflammation, is another mediator that facilitates tumor angiogenesis by initiating coagulation pathways (152).

In relation to tumor angiogenesis driven by microbial imbalance, one study reported that an abundance of *E. coli, B. subtilis* and *S. mitis* leads to increased breast cancer cell invasion and angiogenesis (150). This is because of the effects of the different quorum-sensing peptides produced by these bacteria. Similarly, MALT lymphoma is related to increased levels of *H. pylori* and *H. heilmannii*, which triggers angiogenesis by interacting with the vascular endothelial growth factor (VEGF) receptor (153). Vascular endothelial growth factor-A (VEGF-A) is a known biomarker of angiogenesis in patients with inflammatory bowel disease (150).

**Figure 2:**
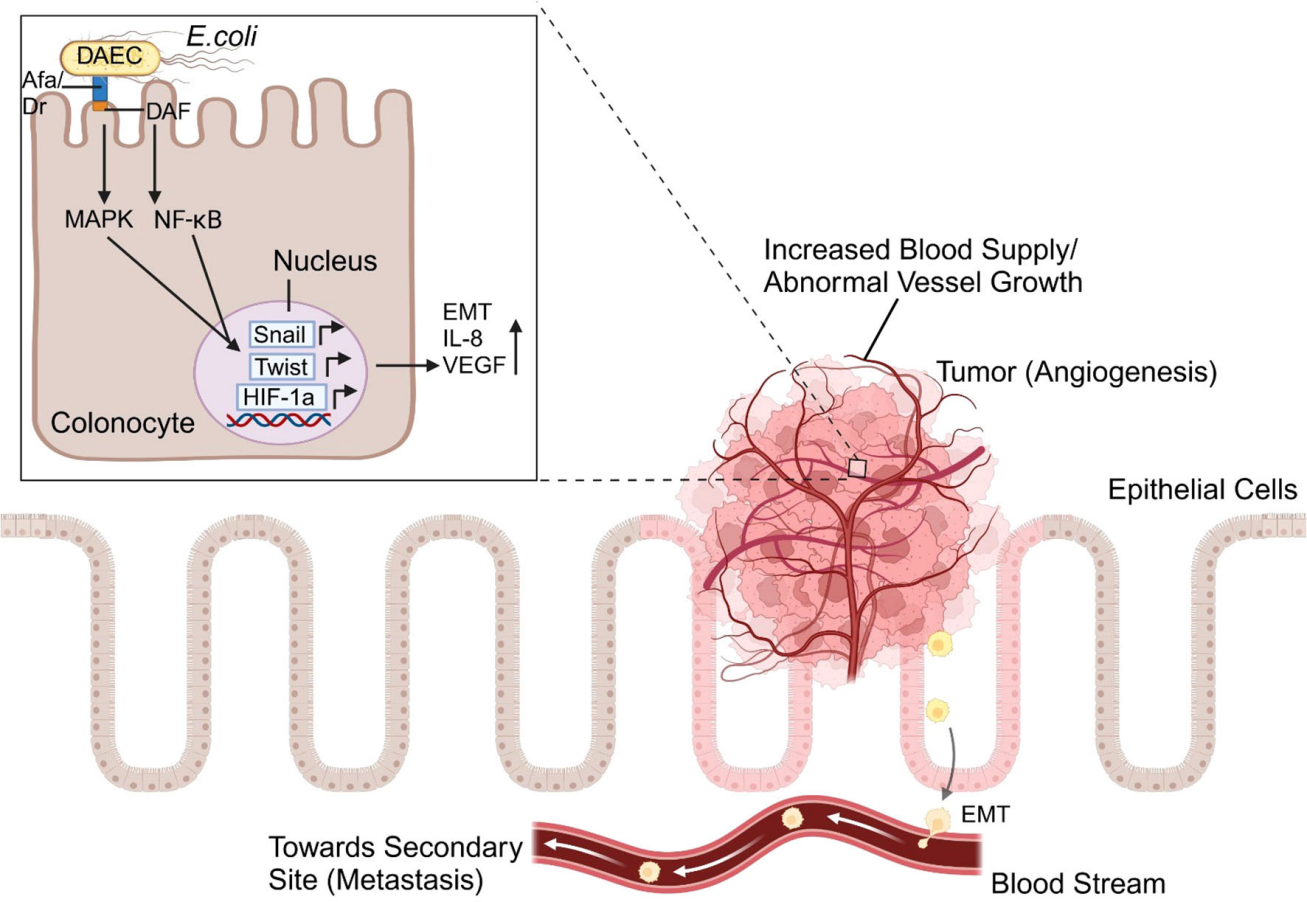
The angiogenesis pathway triggered by Afa/Dr diffusely adhering E. coli (DAEC) begins with bacterial adhesion to intestinal epithelial cells via specific surface receptors like Decay-Accelerating Factor (Daf). This interaction activates intracellular signaling cascades, i.e. the MAPK and NF-κB pathways, which increase the transcription of hypoxia-inducible factor-1 alpha (HIF-1α). This induces the expression of vascular endothelial growth factor (VEGF) and pro-inflammatory cytokines like IL-8. These molecules collectively drive inflammation, promote epithelial-mesenchymal transition (EMT) through the upregulation of Snail and Twist pathways, and stimulate angiogenesis, supporting tumor progression.

In gastric cancer, there are five main biomarkers of tumor-related angiogenesis: the VEGF, angiopoietin (ANG)/endostatin, ANG-like, interleukin (IL), and HIF families (154). *H. pylori*, which is associated with gastritis and increased cancer risk, secretes VacA toxin, a virulence factor that upregulates VEGF expression in the epithelial cells of the gut, activating the epithelial growth factor receptor (EGFR), mitogen-activated protein kinase (MAPK), and COX-2 pathways (150). HIF-1α transcription factor that interacts with VEGF is activated by *H. Pylori* infection. As demonstrated in **Figure 2**, *E. coli* can also activate the HIF-1α transcription factor through the expression of *afa-1* operon, and is linked to colorectal cancer and tumor growth (150). In gastric cancer, the levels of the inflammatory chemokine IL-8 are elevated, which is linked to the growth of tumor blood vessels. COX-2, another biomarker, increases *Bcl-2* expression and triggers *Akt* activation, which results in tumor invasion and angiogenesis (150).

Microbial metabolites interact with the tumor microenvironment through metabolite-cell interactions mediated by specific receptors and transporters. These interactions can be positive (tumor suppressive) or negative (tumor advancing) depending on the type of metabolite (155).

##### 4.2.2.1 Short chain fatty acids

Short chain fatty acids (SCFAs) such as propionate, butyrate, and acetate produced as a result of poly-carbohydrate (starch and fiber) fermentation interact with cells through GPR41, GPR43, and GPR109A receptors and generally have a suppressive effect on carcinomas and adenomas by inhibiting their proliferation. This is especially true for butyrate, which inhibits cancer cell proliferation via the Warburg effect (156). In colorectal cancer (CRC) and hepatocellular carcinoma (HCC) patients, low levels of SCFAs in their stool indicate a low level of butyrate-producing bacteria (e.g., *F. prausnitzii*, *B. uniformis*, *B. vulgaris, Roseburia*, and *Lachnospiracea* species) (1, 3). Propionate and butyrate induce anti-inflammatory effects through the activation of GPR43 and GPR109A receptors, which cause macrophages and dendritic cells to differentiate naïve T-cells into regulatory T-cells (Tregs) that reduce inflammation (157). Although SCFAs have anti-inflammatory and immunosuppressive effects to prevent excessive inflammation during cancer, this could lead to cancerous cells evading the immune response and result in tumor progression. Previous studies have shown that a low concentration of butyrate favors the differentiation of Tregs, which is why it is important to have diverse gut microbiota (157, 158).

##### 4.2.2.2 Secondary bile acids

Bile acids that escape into the colon as a result of high fat intake are converted by colonic microbes into secondary bile acids (e.g. dihydroxycholanoic acid (DCA) and lithocholic acid (LCA)). DCA is a well-studied promoter of carcinogenesis and tumor growth in mammary epithelial cells (144, 159, 160). It functions by activating cell signaling pathways through protein kinase C (PKC) and extracellular signal-regulated kinases 1 and 2 (ERK1/2), driven by epidermal growth factor receptors (EGFR), which may lead to uncontrolled cell growth (155). Moreover, in HCC, secondary bile acids were found to activate the receptors FXR and TGR5, causing Ly6C^low^ monocytes to accumulate inside the liver and differentiate into anti-inflammatory macrophages, which may encourage tumor growth as a result of immunosuppression (157). One study reported that stopping the conversion of primary bile acids to secondary bile acids results in the repression of liver cancer (102). This is because secondary bile acids decrease the expression of the chemoattractant CXCL16, which attracts natural killer cells (NKC) into the liver to destroy and stop tumor progression. This was supported by another study in which mice were colonized with bile acid-metabolizing bacteria, which resulted in decreased numbers of NKCs and tumor growth (157).

##### 4.2.2.3 Polyamines

Polyamines are nitrogen-rich, charged molecules that contribute to healthy cell growth and proliferation, if present in normal amounts. However, abnormal levels of polyamines (e.g., putrescine, spermidine, spermine, and cadaverine) are associated with colorectal cancer, in which the metabolic pathway becomes dysregulated, preventing the breakdown of polyamines, leading to their buildup and abnormal cell proliferation (161). Fecal analysis of patients shows increased levels of amino acids and polyamines (162). Moreover, polyamines prevent the differentiation of macrophages into M1 pro-inflammatory macrophages, which are crucial for attacking and eliminating cancer cells. This occurs through the enzyme ornithine decarboxylase (ODC), which plays a role in the synthesis of putrescine. Putrescine alters the structure of chromatin and prevents the transcription of inflammatory genes required for macrophages to differentiate into the pro-inflammatory M1 type. As a result, the body’s immune response against the tumor is weakened, allowing it to proliferate. High polyamine levels in CRC also impair anti-tumor immune responses by reducing the expression of adhesion molecules, such as CD44 and LFA-1, as well as by decreasing the production of key cytokines, such as IFN-γ and TNF. These changes contribute to the immunosuppressive environment of TME (156).

##### 4.2.2.4 Other metabolites

Some microbes have been shown to convert ethanol into acetaldehyde, a substance that damages DNA and is a significant carcinogen. Another metabolite, hydrogen sulfide, produced by sulfate-reducing bacteria, is toxic to colon cells and promotes their proliferation by activating the ERK1/2 signaling pathway. It also causes DNA damage through the generation of reactive oxygen species (ROS), contributing to inflammation and carcinogenesis in the TME (155). *Lactobacillus* species convert dietary tryptophan into indoles, which activate the aryl hydrocarbon receptor (AhR) in the tumor microenvironment. This activation influences T cells and tumor-associated macrophages (TAMs), which play a role in immune regulation in pancreatic ductal adenocarcinoma (PDAC). The connection between tryptophan metabolism and tumor progression was highlighted when the removal of tryptophan from the diet reduced AhR activity in TAMs. This reduction led to an increase in the infiltration of pro-inflammatory, tumor-fighting CD8+ T cells producing TNFα and IFNγ, suggesting that tryptophan metabolism by *Lactobacillus* can modulate immune responses in PDAC, potentially affecting tumor growth (143).

## 5 Gut dysbiosis and cancer progression

### 5.1 Metastasis and role of microbiota

Gut microbial dysbiosis is not confined to cancer initiation, but also plays a role in cancer progression and metastasis in different types of cancer. Metastasis is a defining feature of malignancies and a major contributor to cancer-related death. For this, the tumor cells must first detach from the primary tumor and enter the bloodstream, followed by the adhesion of these circulating cells to the walls of blood vessels and cell proliferation at the new site (163). The microbiome can do so in three ways: by reshaping the primary tumor microenvironment (TME), by facilitating pre-metastatic niche formation (PMN), and by stimulating epithelial-to-mesenchymal transition (EMT) (145).

Investigating the gut and lung microbes from individuals with non-small cell lung cancer (NSCLC) and healthy individuals revealed an altered composition of both microbiota and an abundance of *Pseudomonas aeruginosa* in individuals with brain metastasis (164). The use of broad-spectrum antibiotics is one of the reasons for gut microbial dysbiosis and defective T-cell function, which can lead to non-small cell lung cancer (NSCLC) and its metastatic spread (165). This argument was also supported by a study in which specific pathogen-free (SPF) mice showed enhanced lung cancer metastasis when treated with broad-spectrum antibiotics owing to the impact of the gut microbiome on circRNA/miRNA expression in the tumor microenvironment (166).

Another study showed that antibiotic-induced gut dysbiosis was linked to metastatic prostate cancer in mice and humans, as antibiotic exposure increased *Proteobacteria* in the gut and activated the NF-κB-IL6-STAT3 pathway, leading to cancer progression and spread (167). An altered microbial state is also involved in endometrial cancer (EC). *Ruminococcus* sp. N15.MGS-57 was enriched in fecal samples of EC patients, in addition to increased levels of fatty acids in the blood, leading to the conclusion that gut dysbiosis can prompt EC metastasis by influencing fatty acid metabolism (168).

Alterations in gut microbes contribute to metastatic niche formation, such as colonization of enterotoxic *B. fragilis* (ETBF) in the gut, which triggers systemic inflammation by enhancing the production of tumor-promoting and inflammatory cytokines (IL6, IL10, IL17A, IL17E, and IL27p28), establishing a pre-metastatic niche in other organs. This, along with immune suppression, fosters a pro-metastatic effect, where breast cancer spreads towards the lungs and liver (169). According to evidence from another study, the pathogenic presence of BTF-producing *B. fragilis* in the gut or mammary duct facilitates the progression and metastasis of breast cancer (170, 171). Mice that were administered an antibiotic mixture and then infected with ETBF via the oral route showed increased cancer progression compared with control mice. Moreover, RNA-sequencing results demonstrated upregulation of genes associated with cell migration, cytoskeletal remodeling, cell invasion, and embryonic pluripotency in the BFT-exposed MCF7 breast cancer cell line. Compared to the RNA-seq data of the control group, BFT-treated MCF7 cells showed enhanced expression of genes involved in the β-catenin and Notch1 pathways (170).

Microorganisms residing in the gut have also been linked to the metastasis of breast cancer to the bone (172). Decreased microbial diversity and diminished populations of *Akkermansia* and *Megamonas* were observed in fecal samples of patients with breast cancer with bone metastasis when compared to healthy individuals and breast cancer patients without metastasis (173). Similarly, research on syngeneic mouse models of hormone receptor-positive (HR^+^) breast cancer demonstrated commensal dysbiosis in the gut, which is responsible for metastatic dissemination of cancer cells to the lungs and lymph nodes and for establishing a pre-metastatic niche by causing inflammation, infiltration of myeloid cells, and fibrosis (172, 174).

Perhaps the most evident impact of gut microorganisms on metastasis can be seen in colorectal cancer (CRC), which can spread to organs such as the lungs, liver, brain, and bones and is mechanistically best described by the ‘seed and soil’ hypothesis. Metastasis relies on the compatibility of cancer cells (seeds) that detach from the primary tumor and settle into a suitable new site (soil), where the microenvironment is conducive to cancerous growth (145). *Fusobacterium nucleatum* (*Fn*) is prominently linked to colon cancer metastasis to the liver (175) and lymph nodes (176). *F. nucleatum* activates the β-catenin pathway by increasing the expression of TLR4 and P-PAK1 proteins (177), the latter being associated with metastatic progression of colorectal carcinomas (178). This bacterium also expresses a lectin, Fap2, which can bind to Gal-GalNAc, a polysaccharide overexpressed in CRC metastasis (179). Oral administration of *F. nucleatum* to mice led to altered intestinal microflora and increased CRC liver metastasis, along with elevated plasma levels of inflammatory immune components (IL9, IL12, IL17A, MCP-1, CXCL1, IFN-γ, and TNF-α). Increased infiltration of myeloid-derived suppressor cells and regulatory T-cells and a decrease in T helper-17 cells and natural killer cells showed the modulatory effect of *Fn* on liver immunity and establishment of a pre-metastatic niche (180).

*Fn* can target cancer stem cells (CSCs) to induce metastasis. *Fn* binds to colorectal cancer stem cells (CR-CSCs) by targeting the Gal-GalNAc molecule on the cancer cell surface with the aid of a docking protein, carcinoembryonic antigen (CEA)-related cell adhesion molecule 1 (CEACAM-1), which is also present on CSCs. This binding stimulates p42/44 MAP activation, eventually leading to cancerous growth and spread (181). Enteric microbes also influence metastasis by interacting with the enteric nervous system. Isovalerate is a gut microbial metabolite that can control serotonin (5-hydroxytryptamine) production by enteric serotonergic neurons. Increased 5-HT initiates Wnt/β-catenin, a pathway responsible for the self-replication capability of cancer stem cells and subsequent CRC progression and metastasis (182).

*Escherichia coli* has been implicated in the metastasis of colorectal cancer to the liver, as it disrupts the gut vascular barrier, translocates to the liver, and fosters pre-metastatic niche formation. In addition, plasmalemma vesicle-associated protein-1 (PV-1) has been identified as a biomarker for increased vascular permeability and the development of metachronous distant metastases (183). To explore the effect of antibiotic-induced intestinal dysbiosis on colorectal cancer liver metastasis (CRLM), different antibiotics were administered to CRC mice, their microbial communities were compared using 16S rDNA sequencing of fecal samples, and immunohistochemistry analysis was performed on liver samples. Results identified enriched levels of *Proteus mirabilis* and *Parabacteroides distasonis*, reduced numbers of Kupffer cells (KCs) and greater liver metastasis in vancomycin-treated mice (184).

Epithelial-mesenchymal transition (EMT) is also responsible for CRC metastasis, a process in which epithelial cells lose their typical features such as cell-cell contact and polarity and become more invasive and mobile, adopting mesenchymal characteristics to better attach to blood vessels and spread through the bloodstream (145, 185). GI bacteria, such as *Fusobacterium nucleatum, Escherichia coli*, enterotoxigenic *Bacteroides fragilis, Salmonella enterica* and *Enterococcus faecalis* are known to cause CRC metastasis via EMT (186). *F. nucleatum* is known to be involved in colorectal cancer (CRC) and colitis-associated colorectal cancer (CAC) progression and metastasis through EMT by triggering the production of neutrophil extracellular traps (NETs) by activating the Toll-like receptor 4 (TLR4)-reactive oxygen species (ROS) and NOD1/2 pathways (187) or by triggering epidermal growth factor receptor (EGFR) signaling (188). A decrease in epithelial marker proteins (E-cadherin) and an increase in mesenchymal marker proteins (Vimentin and N-cadherin) indicated epithelial-mesenchymal transition in CRC tumor cells trapped by NETs (187). Several other markers have been identified for *F. nucleatum*-induced CRC metastasis. For example, elevated expression of endogenous retroviral-associated adenocarcinoma lncRNA (*EVADR*) has been observed in primary CRC tumors that metastasize to non-metastasized ones (189). In cancer cells, the microRNA miR-122-5p acts as a tumor suppressor; however, *Fn* infection causes miR-122-5p downregulation in CRC cells and promotes its release into the bloodstream via exosomes (190). Decreased miR-122-5p expression along with activation of the TGF-β1/Smad signaling cascade leads to epithelial-to-mesenchymal transition and promotes CRC metastasis (191).

### 5.2 Dysbiosis-related resistance to immunotherapy

The tumor’s sensitivity towards targeted treatments such as immunotherapy depends on several factors, including tumor heterogeneity, mutational status of the cells, and extrinsic factors such as host age, genetic predispositions, metabolism, diet, and microbiota. The gut microbiome plays a complex role in this regard: it can generate resistance to cancer immunotherapies as well as mitigate resistance (192). Dysbiotic gut conditions create resistance to immune checkpoint inhibitors (ICIs) (193). Reduced diversity of microbial communities in the gut and abundance of *Ruminococcus gnavus*, *Bacteroides massiliensis, Bacteroides dorei, Bacteroides ovatus* and *Blautia producta* are associated with shorter progression-free survival in melanoma patients undergoing immunotherapy (194). The commensal *Akkermansia muciniphila* has been established as a biomarker for a beneficial response to immunotherapy in non-small cell lung cancer. However, antibiotic-induced gut dysbiosis can cause excessive proliferation of *A. muciniphila* and *Clostridium*, which can lead to resistance to therapy (195). A clinical study where fecal samples of patients with advanced renal cell carcinoma (RCC) were analyzed revealed that the gut microbial composition was significantly altered by the use of antibiotics and tyrosine kinase inhibitors, in turn creating primary resistance to the nivolumab (anti-PD-1) immunotherapy (196). Metagenomic sequencing and taxonomic profiling from samples of 65 people with hepatobiliary cancers concluded that enriched levels of Veillonellaceae family were correlated with resistance to anti-PD-1 therapy and a lower chance of progression free survival (197). In most of the cases, this dysbiosis is induced by antibiotics or drugs given to manage cancer-related symptoms such as acid reducers, corticosteroids and anxiolytic drugs (198).

CpG-oligonucleotides (CpG-ODNs) are another form of immunotherapy that works by mimicking bacterial DNA, targeting the Toll-Like Receptor-9 (TLR9), and triggering an immune response through T cells, NK cells, B cells, macrophages, dendritic cells, and cytokine release in the host that can simultaneously attack tumor cells (199). Research shows that an intact gut commensal population is needed for efficient CpG-oligonucleotide therapy and platinum chemotherapy (200). Disruption of bacteria due to antibiotic treatment leads to reduced tumor necrosis, lower levels of cytokines, and less ROS production, causing poor treatment response of myeloid-derived cells in the tumor microenvironment (201).

Studies have suggested that gut bacteria are associated with resistance to other forms of cancer therapies as well. One such bacterium is *Fusobacterium nucleatum* which is associated with adverse prognosis and recurrence after chemotherapy (using 5-fluorouracil and oxaliplatin) for colorectal cancer (CRC), which influences molecules such as TLR4 and MYD88, and triggers microRNAs and autophagy mechanisms (56, 202, 203). Analogous findings were observed in the case of esophageal squamous cell carcinoma (ESCC), where a higher amount of intratumoral *F. nucleatum* was linked to a poor response to neoadjuvant chemotherapy (NAC) based on docetaxel, 5-FU, and cisplatin regimens prior to esophagectomy (204).

The intratumoral presence of a class of bacteria, Gammaproteobacteria, causes resistance to the chemotherapeutic drug gemcitabine, which is usually used in the treatment of pancreatic ductal adenocarcinoma (PDAC). These bacteria have the enzyme cytidine deaminase (CDD_L_), which converts the active form of gemcitabine into its inactive form (2′,2′-difluorodeoxyuridine), rendering it unable to inhibit DNA synthesis in tumor cells (205,206). Although the study did not directly indicate the gastrointestinal origin of the bacteria, it is plausible to infer that these bacteria can translocate from the gut to the tumor due to a dysbiosis-induced leaky gut.

## 6 Diagnostic approaches

### 6.1 Non-invasive diagnostic tools for dysbiosis assessment

Tissue and stool samples are typically analyzed to assess gut dysbiosis in individuals. However, since endoscopic biopsy of intestinal tissue is an invasive procedure and carries the risk of infection and discomfort to the patient, stool samples are preferred for determining diversity and distribution of microbes in the gut and for dysbiosis assessment, although this has its own limitations (207, 208). In metagenomics and meta-transcriptomics, sequencing technologies such as shotgun sequencing or 16S rRNA sequencing are applied to determine microbial diversity in samples, and in metabolomics (the assessment of microbial metabolomics markers in the gut) different approaches such as the oral carnitine challenge test or nuclear magnetic resonance (NMR) technology are used. Moreover, urine and hydrogen/methane breath tests are also used to check for dysbiosis. The measures and metrics used to quantify dysbiosis are referred to as the dysbiosis indices.

#### 6.1.1 Meta-genomics and meta-transcriptomics

Metagenomics is the study of the entire genome of microbes present in a sample. 16S rRNA sequencing is a method employed where the hypervariable regions of microbial DNA (e.g., 16S rRNA gene) are sequenced to identify different species within a sample (209, 210). Conversely, the shotgun method sequences the entirety of the DNA to identify rarer microbial species. Researchers have extensively applied these approaches in studies of gut dysbiosis, particularly in colorectal cancer (CRC) (211). For example, one study employed a combination of 16S rRNA sequencing of fecal samples with clinical risk factors (age, race, and BMI) to enhance the diagnostic accuracy in distinguishing healthy individuals from those with adenomas and carcinomas (212). Similarly, in another study, 20 microbial gene markers that distinguished CRC from control microbiomes were identified, validating key markers such as *Peptostreptococcus anaerobius* and *Fusobacterium nucleatum* across multiple international cohorts. These microbial gene markers have shown potential for early CRC diagnosis, even in stages I-II (213).

Meta-transcriptomics, which uses RNA sequencing to analyze the active gene expression (mRNA) of microbes, can be integrated with metagenomics to provide a complete picture of microbial diversity and functional activity. These methods can be used to compare the gut microbiota of healthy individuals with those suffering from gut dysbiosis (207).

#### 6.1.2 Metabolomics

The metabolites produced by gut bacteria have been studied to assess gut dysbiosis. Dysbiosis induces changes in the metabolite profiles of patients relative to those of healthy individuals; therefore, they can be used as biomarkers for diagnosis. Advanced tools, such as ultra-high-performance liquid chromatography-tandem mass spectrometry (UHPLC-MS) and proton nuclear magnetic resonance spectroscopy (^1^H NMR), are used to study these metabolites. Additionally, oral carnitine challenge tests are performed to identify certain metabolites (207).

NMR has proven to be particularly effective in profiling metabolites from patients with CRC. This approach, combined with statistical techniques such as principal component analysis (PCA) and orthogonal partial least squares discriminant analysis (OPLS-DA), allows the comparison of metabolite profiles between patients and healthy controls. In studies profiling the fecal metabolites of CRC patients across different stages of the disease, OPLS-DA was able to clearly distinguish between CRC patients and healthy controls, based on their unique metabolomic signatures (211). CRC patients exhibited depleted levels of beneficial metabolites, such as acetate, butyrate, propionate, glucose, and glutamine, while increased levels of metabolites, such as succinate, proline, alanine, dimethylglycine, valine, glutamate, leucine, isoleucine, and lactate, were observed. Microbiome-derived metabolites including lipopolysaccharides, SCFAs, secondary bile acids, and tryptophan-related metabolites play critical roles in the pathology of dysbiosis and CRC development. These metabolites can be measured noninvasively from various biological samples, including cerebrospinal fluid (CSF), plasma, urine, and feces, using NMR spectroscopy (211). High-performance liquid chromatography-mass spectrometry and gas chromatography–mass spectrometry are typically used to check for disruption of tryptophan metabolism. Tryptophan metabolites are associated with a healthy gut (207).

#### 6.1.3 Hydrogen/methane breath test

This test identified dysbiosis by measuring the degree of microbial fermentation in the gut. This is done by measuring the amount of methane or hydrogen released from the breath of a person after consuming a substrate solution, such as lactulose, which is digestible by bacteria. The steady increase in the readings of this test indicates possible dysbiosis in the gut (207).

#### 6.1.4 Urine test

This is also called the mannitol-lactulose intestinal permeability test, in which the integrity of the intestinal lining is evaluated following the consumption of lactulose or mannitol. A leaky gut caused by dysbiosis results in elevated levels of these sugars present in the urine of an individual (207).

### 6.2 Biomarkers of gut dysbiosis and cancer progression

Biomarkers are used to assess the impact of a given treatment on the presence or progression of a specific disease. Dysbiosis markers are identified by analyzing microbial diversity through alpha diversity (within-sample diversity), beta diversity (between-sample differences), and gamma diversity (overall diversity across environments) using metrics such as the Shannon Index, Simpson’s Index, Chao-1 Index, and Bray-Curtis Distance (207, 209). Thus, a range of different biomarkers have been identified in association with dysbiosis and the progression of different types of cancers.

For instance, one validated biomarker for colorectal cancer is the bacterium *Fusobacterium nucleatum*, specifically the subspecies *F. vicentii* and *F. animalis*, which have been found in higher concentrations in fecal samples of colorectal cancer (CRC) patients than in healthy individuals (214). *Peptostreptococcus stomatis*, *Parvimonas*, and *Porphyromonas asaccharolytica* are also significantly enriched in early-stage CRC patients (214, 215). Additionally, specific genes, such as transposases from *Peptostreptococcus anaerobius* and butyryl-CoA dehydrogenase from *Fusobacterium nucleatum* have been confirmed as biomarkers in European cohorts for CRC detection. Another potential marker, the *m3* gene from *Lachnoclostridium* spp., has shown promise for early CRC diagnosis, with notable differences observed across various stages of the disease (214, 216). *E. coli* that produces colibactin and *B. fragilis* that secrete the Bacteroides fragilis toxin (BFT) have been associated in multiple studies with TNM classified aggressive cancer stages (217, 218).

In patients with pancreatic ductal adenocarcinoma (PDAC), Proteobacteria, *Pseudomonas* and *Elizabethkingia* are abundant (219). Similarly, greater populations of *Sutterella, Veillonella, Bacteroides, Odoribacter*, and *Akkermansia* have been observed in the fecal samples of patients with pancreatic cancer than in healthy controls (114, 220). DNA from *Helicobacter pylori* has also been detected in as much as 48% of pancreatic tumor samples, indicating a possible association between the bacterium and pancreatic cancer (221).

Another study identified specific microbial signatures linked to different breast cancer types (222). For endocrine receptor-positive cancer, *Arcanobacterium* and *Bifidobacterium* bacteria, *Mucor* fungi, and the parasite *Brugia* have been linked. In Her2-positive cancer, Streptococcus bacteria, *Epidermophyton* fungi, and *Balamuthia* parasites were found. Triple-positive cancers showed connections to *Bordetella*, *Penicillium* fungi, and *Ancylostoma* parasites. *Aerococcus* bacteria, *Alternaria* fungi, and *Leishmania* parasites were identified in triple-negative breast cancer. These findings highlight the potential of microbial biomarkers in gut dysbiosis and cancer diagnosis.

## 7 Therapeutic strategies

### 7.1 Modulating gut microbiota for enhanced immunotherapy response

Among the different methods used to treat and manage cancer, such as radiation and chemotherapy, immunotherapy is a way in which one’s own immune system is strengthened to fight cancer. A vast range of strategies, including cancer vaccines, monoclonal antibodies, cytokine therapy, immune checkpoint inhibitors (ICIs), oncolytic viruses, and CAR T-cell therapy, are used in cancer immunotherapy (223). It has been established in earlier arguments that gut microbiota can modulate immune processes in the host; hence, it is also implicated in controlling the therapeutic response, toxicity, and efficacy of cancer therapies (200).

**Figure 3:**
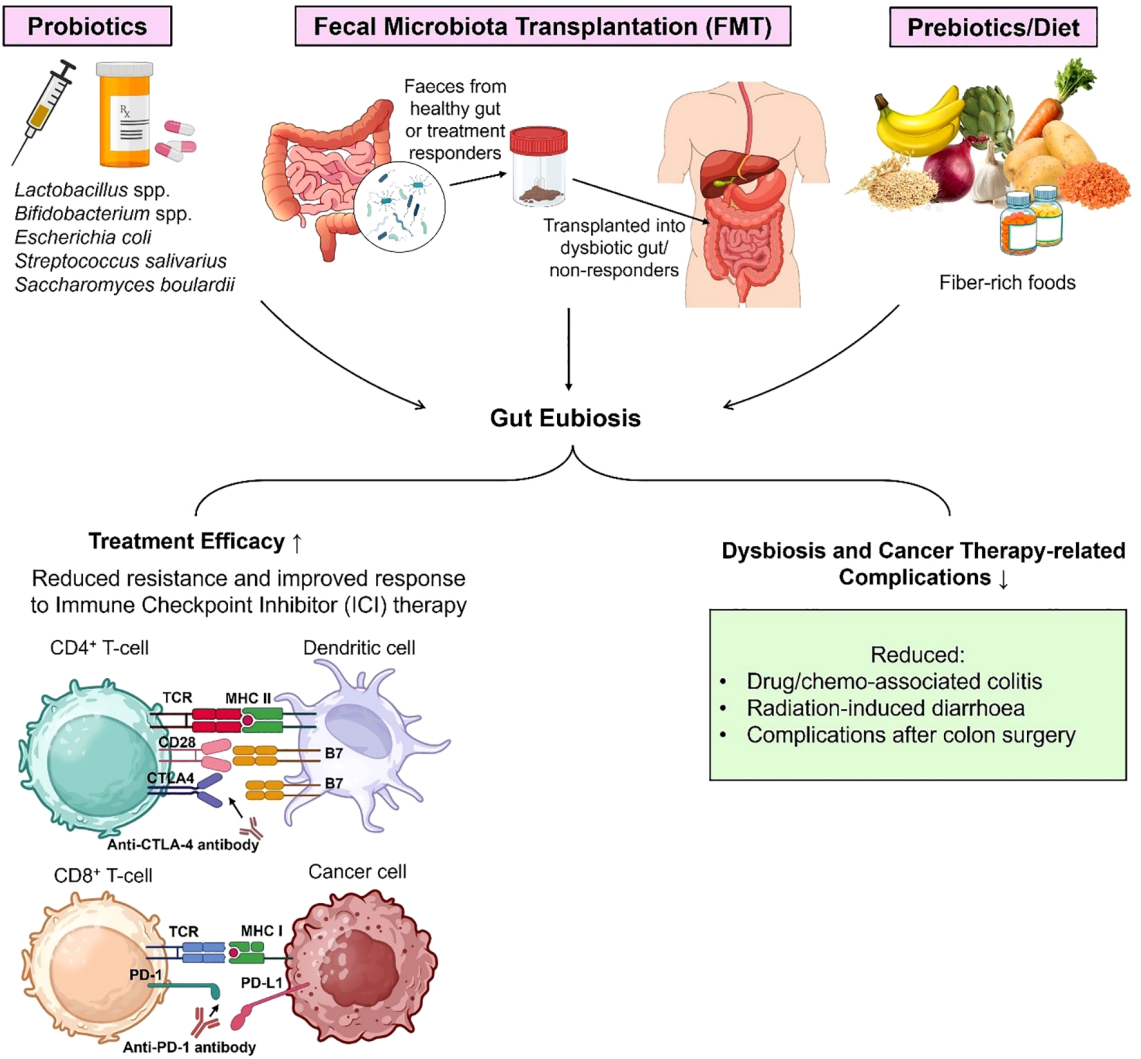
Gut microbiota dysbiosis can be managed and prevented in several ways to reduce the risk of developing dysbiosis-induced cancer. Probiotic treatments (live single strain bacteria or bacterial consortia), prebiotic and dietary interventions, and fecal microbiota transplantation (FMT) can help reverse and avoid dysbiosis. FMT involves transplanting fecal/gut bacteria from a healthy individual to a host with dysbiosis to restore their microbial balance (eubiosis). These strategies not only enhance the efficacy of chemotherapy and immune checkpoint inhibitor (ICI) therapy (anti-CTLA-4 and anti-PD-1/anti-PD-L1) but also reduce complications associated with cancer therapy such as colitis and diarrhoea caused by chemo and radiation.

Cancer cells use checkpoint proteins such as PD-L1/PD-1 and CTLA-4 to turn off the T-cell response. Immune checkpoint inhibitors block these proteins and act as antitumor drugs (144). **Figure 3** illustrates how these checkpoint inhibitor antibodies work by binding to their target receptors on immune cells. A study comparing melanoma growth between two groups of genetically similar mice with different gut microbes found that *Bifidobacterium* presence was linked to an antitumor response. They also found that administering *Bifidobacterium* through oral gavage and programmed cell death ligand- 1 (PD-L1)–specific antibody therapy had the same effect on tumor growth reduction (224). Similar results have been reported in human studies. Analysis of the fecal microbiome of 112 melanoma patients undergoing anti–programmed cell death 1 protein (PD-1) immunotherapy showed that patients who responded well to therapy had increased levels of Ruminococcaceae bacteria and an overall higher alpha diversity of microbes than non-responders (225). Another study revealed an abundance of *Collinsella aerofaciens, Bifidobacterium longum* and *Enterococcus faecium* in patients who responded to anti–PD-1 immunotherapy for metastatic melanoma compared with those who did not respond to treatment (226).

The diversity and composition of the gut microbiome are also associated with treatment efficacy in non-small cell lung cancer (NSCLC). When researchers performed 16S rRNA sequencing of fecal samples of NSCLC patients receiving the PD-1 blockade antibody nivolumab, they found that those responsive to the treatment had greater microbial diversity and an abundance of *Bifidobacterium longum*, *Alistipes putredinis* and *Prevotella copri*. These individuals also had a higher number of natural killer cells and memory CD8^+^ T cells and a longer progression-free survival (227). Experiments on other cancer types have yielded similar results; for instance, a study on hepatocellular carcinoma (HCC) patients described an elevated abundance of *Akkermansia muciniphila* and *Ruminococcaceae* spp. in the intestines to be linked with an improved response to anti-PD-1 immunotherapy (228).

The bacterial species *B. fragilis* and *B. thetaiotaomicron* have been associated with improved antitumor effects of anti-cytotoxic T-lymphocyte-associated antigen-4 (CTLA-4) therapy in melanoma patients. This was confirmed when mice administered fecal microbiota transplantation of *B. fragilis* from melanoma patients overcame CTLA-4 blockade resistance (229). Intestinal bacteria have also been shown to reduce cancer therapy-related complications and inflammatory effects of cancer drugs. The fecal microbiome was analyzed in patients with metastatic melanoma before and after ipilimumab (anti-CTLA-4) treatment. Patients whose samples showed greater enrichment of the Bacteroidetes phylum were more resistant to the development of immune-induced colitis (230). The same was evidenced by another study that showed the presence of *Bacteroides* in melanoma patients receiving ipilimumab therapy to be associated with resistance to drug-induced colitis, and the abundance of *Faecalibacterium* was associated with longer progression-free survival (231). Immune checkpoint inhibitors have been a successful therapy for certain malignancies, but work is still underway to devise methods to reduce therapy-induced toxicities, including intestinal inflammation, in patients (232). Therefore, the identification of gut microbial signatures for resistance or toxicity to immunotherapy is crucial.

Bacteria such as *Lactobacillus johnsonii, Bifidobacterium pseudolongum* and *Olsenella* sp. have been shown to increase the efficacy of both PD-L1 and CTLA-4 immune checkpoint blockade (ICB) therapy in four types of cancers in mice. Moreover, it was observed that mice monocolonized with *Bifidobacterium pseudolongum* had a bacterial metabolite named inosine present in their sera, which helps with antitumor T cell activation (233). Scientists have identified an 11-strain bacterial consortium isolated from healthy human fecal samples that can enhance ICIs efficacy by stimulating interferon-γ (IFN-γ)-producing CD8+ T-cells in adenocarcinoma and melanoma mouse models (234). These include *Alistipes senegalensis, Eubacterium limosum, Phascolarctobacterium faecium, Ruminococcaceae bacterium cv2*, *Parabacteroides* spp., *Fusobacterium ulcerans* and five *Bacteroides* spp (234, 235). Upon colonizing mice with the 11-strain mixture, a significant reduction in tumor growth was observed in mice receiving anti-PD-1 therapy or anti-CTLA-4 therapy (234). Metagenomic shotgun sequencing of stool specimens of metastatic melanoma patients has shown enriched levels of *Bacteroides caccae* in people responsive to different immune checkpoint inhibitor treatments, including ipilimumab (anti-CTLA-4), nivolumab (anti-PD-1), a combination of ipilimumab and nivolumab, and pembrolizumab (anti-PD-1) (236).

#### 7.1.1 Immunometabolism reprogramming for reversing immunotherapy resistance

Immune cells are capable of altering their metabolic processes in response to environmental cues. Cancer cells and their tumor microenvironment (TME) exploit these metabolic signals to evade immune response. Warburg metabolism is a signature sign of carcinogenesis, where cancer cells consume glucose via aerobic glycolysis and produce lactate even when oxygen is present (237). This differs from the metabolism of normal mammalian cells which usually metabolize glucose via oxidative phosphorylation when oxygen is present and shift to glycolysis only in anoxic conditions (237, 238). Changes in the TME such as lactate accumulation and hypoglycemia, hinder the functioning of T-cells, dendritic cells and macrophages, the same reason which leads to the failure of cancer immunotherapies (238). Gut microbiota closely interacts with the immune system, playing a role in shaping tumor immune microenvironment (TIME) and has known roles in immunotherapy efficacy as well (14, 239). Therefore, immunometabolic reprogramming by modulating gut microbiome has emerged as a target for countering resistance to immunotherapy.

Cancer cells compete with surrounding cells for nutrients. Increased expression of methionine transporter SLC43A2 on tumor cell epigenetically disrupts methionine metabolism in CD8^+^ T cells. It inhibits dimethylation at lysine 79 of histone H3 (H3K79me2) and decreases STAT5 expression, lowering the cytotoxic potential of T cells (240, 241). Microbes having the potential to affect methionine metabolism can be tested to see if they can restore T cell function as reprogramming of tumor methionine metabolism has been identified as a potential therapeutic source for hepatocellular carcinoma (242). Similarly, when CD4^+^ helper T cells compete with tumor cells and myeloid cells for polyamine uptake, the polyamine deficiency can cause problems with differentiation of T cells into subtypes (T_H_1, T_H_2, T_H_17, and T_regs_), promoting immunosuppression and tumor growth (243). Gut bacteria which produce polyamines as metabolites can be used to restore the functional metabolic state of T cells.

Metabolites produced by gut microbiota such as SCFAs can boost oxidative phosphorylation in Tregs and induce T cell differentiation into effector cells T_H_1 and T_H_17, needed for antitumor activity. Gut microbes can also restore the nutrient availability in TME for the metabolic reprogramming of tumor-associated macrophages (TAMs) and prevent immune cell exhaustion (241). Furthermore, certain bacteria and bacterial metabolites can regulate macrophage polarization, changing the proinflammatory M1 state to an anti-inflammatory or pro-tumor M2 state. A recent study has provided compelling evidence in this regard. After antibiotic cocktail caused gut dysbiosis in glioblastoma model, a decrease in SCFAs and increase in M2 macrophages was observed in the TME. However, oral administration of SCFAs triggered M2 to M1 polarization and increased the M1 population by activating glycolysis in TAMs (244). The strain ZY-312 of *B. fragilis* is known to direct macrophages to an M1 phenotype enhancing their phagocytic potential against cancer cells (245).

Lactate-producing gut bacterial species in the gut can also be targeted for immunometabolic reprogramming. Lactate is an oncometabolite (246, 247), which, if present in the TME can drive polarization of M2-like macrophages through the ERK/STAT3 pathway activation, promoting breast cancer growth and metastasis (248). Suppressing this process can have the reverse effect.

### 7.2 Probiotics/prebiotics in cancer therapy

Gut microbes are not only involved in determining the efficacy of cancer therapies (249), but can also be used as probiotics to treat cancers and adverse effects of cancer treatments. For example, oral delivery of gut anaerobic *Bifidobacterium* to mice caused its accumulation in the tumor microenvironment and enhanced the anti-tumor response of anti-CD47 immunotherapy via the stimulator of interferon genes (STING) pathway (250). CD47 is a signaling molecule that is substantially expressed in malignant tumors, allowing tumors to evade macrophage attack by giving a ‘do not eat me’ signal, whereas CD47 inhibition does the opposite (144).

Mouse models have been used to test for new probiotic treatments for hepatocellular carcinoma. Feeding mice with Prohep (a mixture of *Lactobacillus rhamnosus* GG, *Escherichia coli* Nissle 1917 and heat-inactivated VSL#3 in 1:1:1) reduced the production of T_H_17 cells (producing IL-17), upregulated anti-inflammatory Tregs, and downregulated genes responsible for angiogenesis, subsequently shrinking the tumor significantly (251). *Lactobacillus rhamnosus* (Antibiophilus^®^) and *Lactobacillus acidophilus* supplementation has been shown to reduce radiation-induced diarrhea, a common side effect of ionizing radiation in the lower abdomen or pelvic region (252, 253). A pilot clinical study was conducted on 190 patients who were about to undergo adjuvant radiotherapy for rectal, cervical, or sigmoid cancer. The treatment group was administered preemptive doses of VSL#3, a probiotic containing three strains of bifidobacteria (*B. infantis, B. longum*, and *B. breve*), four strains of lactobacilli (*L. acidophilus, L. plantarum, L. delbruekii subsp. Bulgaricus* and *L. casei*), and one strain of *Streptococcus salivarius subsp. thermophilus*. The results revealed that more patients in the placebo group developed radiation-associated diarrhea and gastrointestinal toxicity than those given VSL#3 (254). Commonly used probiotic treatments used along with cancer therapy are summarized in **Figure 3**.

Similar results were obtained when using bacteria to reduce the adverse effects of chemotherapy. The anticancer drug cyclophosphamide (CTX) has a tendency to damage the gut epithelial lining, causing gut bacteria to translocate to secondary lymphoid organs and change gut bacterial composition. A study performed in mice showed *Lactobacillus murinus, Lactobacillus johnsonii*, and *Enterococcus hirae* translocated to the spleen and mesenteric lymph nodes after CTX treatment. Oral administration of *E. hirae* improves CTX efficacy by driving pathogenic T_H_17 antitumor activity and activating tumor-specific memory T_H_1 and cytotoxic T cells (255).

A growing body of literature supports the use of beneficial gut bacteria as preoperative probiotics to reduce the risk of complications after colon surgery for colorectal cancers. A double-blind study conducted on 100 colorectal carcinoma patients showed that the administration of oral probiotic capsules (containing *Lactobacillus plantarum*, *Lactobacillus acidophilus* and *Bifidobacterium longum*) for 6 days before and 10 days after colorectomy improved gut epithelial barrier function by reducing gut permeability and bacterial translocation, and enhancing the expression of proteins in the mucosal tight junctions. Increased diversity of fecal bacteria, decreased presence of enteropathogenic bacteria in the blood, and reduced risk of diarrhea and other infections were observed in the probiotic group compared with the placebo group (256). In another randomized clinical trial conducted on 33 patients who underwent colon resection for colon cancer, patients were administered a 7-day oral preoperative dose of the probiotic fungus *Saccharomyces boulardii.* Postoperative mRNA analysis of tumor tissues revealed lower levels of inflammatory cytokines (IL-10, IL-1β, and IL-23) in mucosal tumor samples in the probiotic group than in the control group (232, 257).

Gut commensals may be involved in the mode of action of certain anticancer compounds. For example, abiraterone acetate (AA) primarily works as an antiandrogenic drug; however, it also affects the gut microbial composition by minimizing bacteria that utilize androgens (*Corynebacterium* spp.) and increasing the beneficial bacteria *Akkermansia muciniphila* in patients with castrate-resistant prostate cancer (CRPC). When AA is combined with androgen deprivation therapy, *A. muciniphila* can increase the synthesis of vitamin K2 (menaquinone), which has anti-tumor properties (258). Oral administration of *A. muciniphila* has been shown to reverse anti-PD-1 resistance in mice models (193). A recent study identified castalagin, a polyphenol extracted from camu-camu (*Myrciaria dubia*) berry, as a prebiotic anticancer compound that can increase the efficacy of anti–PD-1 therapy by interacting with the gut microbiota. Oral administration of castalagin to mice resulted in the enrichment of bacterial species known for enhanced immunotherapeutic response against cancer and CD8^+^/FOXP3^+^CD4^+^ activity. Mechanistically, castalagin binds to the envelope of *Ruminococcus bromii* to induce antitumor activity (259). Previous studies have already shown higher gut microbial diversity and abundance of *Ruminococcaceae* and *Agathobacter*, indicating a higher progression-free survival rate in NSCLC patients treated with anti–PD-1/PD-L1 antibodies (260).

If gut dysbiosis can lead to oncogenic initiation and resistance to cancer therapy, reversing dysbiosis would have an inverse effect. In 2024, a study revealed significant changes in gut microbial composition in murine models of glioblastoma (GBM), and these alterations were attributed to nutrient changes in the gut, particularly tryptophan depletion. Subsequently, dietary supplementation of tryptophan restored beneficial gut bacteria *Duncaniella dubosii*, improved circulation of T-cells, and improved the effectiveness of anti-PD-1 therapy for brain tumors (261). Tumor tissues of colon cancer patients have an altered gut microbiota, especially the overgrowth of bacteria such as *Fusobacterium*, *Peptostreptococcus* and *Selenomonas*. Restoration of microbial homeostasis by probiotic tablets (containing *Bifidobacterium lactis* and *Lactobacillus acidophilus*) preoperatively resulted in a reduction of CRC-associated microbes in fecal samples of the probiotic group compared to the control group (262).

Similarly, certain gut bacteria are hallmarks of metastatic cancers, and the reversal of dysbiosis leads towards better patient outcomes and therapeutic effects. The bacterial metabolite indolepropionic acid (IPA), derived from tryptophan metabolism, exerts cytostatic effects on metastatic breast cancer cells, that is, it inhibits cancer cell proliferation and movement, decreases epithelial-to-mesenchymal transition, and enhances ROS production and lymphocyte infiltration into the tumor without harming non-cancerous cells surrounding the tumors (263). *Salmonella*, a facultative anaerobe known to cause enteric infections, can be modified for use as a neoadjuvant therapy in breast cancer (264) and Lewis lung carcinoma (265). Intracardial injection of *Salmonella typhimurium* A1-R into nude mice inhibited tumor growth and prevented bone metastasis in breast cancer (264). Another microbial metabolite, sodium butyrate, has been shown to positively alter microbial composition in the gut in a mouse model, along with increasing T_H_17 and NK cells and decreasing T regulatory immune cells, which improves host immunity against colorectal cancer liver metastasis (266). Removal of specific gram-positive bacteria in the gut that can metabolize primary bile acids to secondary bile acids generates an anti-tumor effect in liver cancer. The presence of primary bile acids promotes CXCL16 expression in liver sinusoidal endothelial cells, leading to the accumulation of CXCR6^+^ natural killer T cells. NKT cells are regulators of anti-tumor immunity, and this mechanism shows therapeutic potential for liver cancer therapy (102).

### 7.3 Fecal microbiota transplantation for cancer patients

Fecal microbiota transplantation (FMT) is an emerging approach used in cancer therapy to restore the microbial balance in the gut by transferring fecal matter from a healthy host to a host facing dysbiosis, as outlined in **Figure 3** Since the gut microbiota can influence cancer initiation, progression, and efficacy of cancer therapies via different mechanisms, FMT provides a corrective method to deliver beneficial microbes to a host.

Apart from its clinical success in treating *Clostridium difficile* infections (267), it has also been explored in various cancers. In a study of hepatocellular carcinoma (HCC), FMT was used to transfer gut microbiota from responders and non-responder HCC patients undergoing radiotherapy. Researchers have validated in humans as well as mice that gut dysbacteriosis weakens the antitumor immune response by hampering cGAS-STING-IFN-1 signaling in dendritic cells and downregulation of cytotoxic T cells, reducing the response to radiotherapy (268). FMT is also useful for overcoming resistance to immune checkpoint inhibitor therapy. Experiments on antibiotic-treated or germ-free mice administered fecal microbiota transplantation from cancer patients on anti-PD-1 therapy showed resistance to therapy in mice that received FMT from non-responders and effective antitumor activity in mice that received FMT from responders (193, 225, 226, 259). Similar outcomes were documented when the delivery of *B. fragilis* into mice by fecal microbial transplantation enhanced the efficacy of anti-CTLA-4 immunotherapy for melanoma (229).

This promising technique is being tested in clinical trials for patients who have failed PD-1 blockade immunotherapy (NCT03353402) and to prevent dysbiosis-induced complications in cancer (NCT02928523). The latter proposes using autologous fecal microbiota transplantation (AFMT), which involves transplanting one’s own fecal microbiota from a healthier state, to restore the GI tract to its eubiotic state in acute myeloid leukemia (AML) patients who face the adverse effects of chemotherapy and drug resistance. In human trials, FMT is performed via colonoscopy/gastroscopy or administered through frozen or lyophilized pills (232).

The problem lies with the precision of this approach because there is a possibility of transfer of microorganisms with unknown pathogenic potential or unknown effects related to cancer, and the fact that microbial biomarkers for all cancer types are not yet fully known.

## 8 Conclusions and future directions

While there is a robust body of evidence suggesting a relationship between gut dysbiosis leading to chronic inflammation and cancer, the precise molecular and immunological mechanisms by which microbes do so are still not fully understood, especially for non-GI cancers like brain, lung, and breast cancer. Majority of research on gut microbiota in relation to cancer has been focused on bacteria, leaving room for potential research on gut mycobiota (fungi) and viruses that could be involved in oncogenesis. Furthermore, the inter-individual variability in the gut microbiome makes it unclear to what extent existing research can translate its findings to formulate standardized interventions to manage dysbiosis. There is a need for studies on ethnic groups underrepresented in the literature because different communities have different dietary habits and microbiomes.

The link between gut dysbiosis and cancer is a double-edged sword. While certain microbes lead to tumor development, others can strengthen anti-tumor immunity; therefore, investigating the precise microbial signatures is necessary to use them as not only biomarkers for early cancer detection but also for personalized therapies. The heterogeneity of cancer compels us to move towards a more tailored therapeutic approach. Personalized microbial-based probiotic and prebiotic treatments, fecal microbiota transplantation (FMT), and other adjunctive therapies could be an avenue for exploration. Understanding the interactions of gut microbiota with anticancer drugs can provide insights into how microbiota control chemoresistance, resistance to immune checkpoint inhibitors, and the efficacy of cancer therapeutics. Future research could involve longitudinal studies that track changes in gut microbial composition and how they influence patient outcomes pre-, peri-, and post-treatment.

This review discussed the existing work on gut microbiota-cancer interaction, covering aspects from causes of dysbiosis to mechanisms leading to tumorigenesis and metastasis and bacteria associated with different cancers, to diagnosis and therapeutic potential. Research in this field is evolving and offers promising directions for future exploration such as metabolomic profiling in cancer patients to find targets for immunometabolic reprogramming to restore antitumor immunity, and the epigenetic markers and pathways through which some microorganisms lead towards cancer. A deeper mechanistic understanding of how gut microbes influence immunometabolism and therapy response will be critical for harnessing their full therapeutic potential in oncology.
